# Alzheimer's Disease Risk Gene *SORL1* Promotes Receptiveness of Human Microglia to Pro‐Inflammatory Stimuli

**DOI:** 10.1002/glia.24659

**Published:** 2024-12-17

**Authors:** Peter Lund Ovesen, Kristian Juul‐Madsen, Narasimha S. Telugu, Vanessa Schmidt, Silke Frahm, Helena Radbruch, Emma Louise Louth, Anders Rosendal Korshøj, Frank L. Heppner, Sebastian Diecke, Helmut Kettenmann, Thomas E. Willnow

**Affiliations:** ^1^ Max‐Delbrück‐Center for Molecular Medicine in the Helmholtz Association (MDC) Berlin Germany; ^2^ Department of Biomedicine Aarhus University Aarhus Denmark; ^3^ Technology Platform for Pluripotent Stem Cells, Max Delbrück Center for Molecular Medicine in the Helmholtz Association Berlin Germany; ^4^ Department of Neuropathology, Charité – Universitätsmedizin Berlin Corporate Member of Freie Universität Berlin and Humboldt‐Universität Zu Berlin Berlin Germany; ^5^ Department of Neurosurgery Aarhus University Hospital Aarhus Denmark; ^6^ Department of Clinical Medicine Aarhus University Aarhus Denmark; ^7^ Cluster of Excellence, NeuroCure Berlin Germany; ^8^ German Center for Neurodegenerative Diseases (DZNE) Berlin Berlin Germany; ^9^ Shenzhen University of Advanced Technology, Shenzhen Key Laboratory of Immunomodulation for Neurological Diseases Shenzhen China

**Keywords:** Alzheimer's disease, brain inflammation, microglia, SORLA, VPS10P domain receptors

## Abstract

Sorting protein‐related receptor containing class A repeats (SORLA) is an intracellular trafficking receptor encoded by the Alzheimer's disease (AD) gene *SORL1* (*sortilin‐related receptor 1*). Recent findings argue that altered expression in microglia may underlie the genome‐wide risk of AD seen with some *SORL1* gene variants, however, the functional significance of the receptor in microglia remains poorly explained. Using unbiased omics and targeted functional analyses in iPSC‐based human microglia, we identified a crucial role for SORLA in sensitizing microglia to pro‐inflammatory stimuli. We show that SORLA acts as a sorting factor for the pattern recognition receptor CD14, directing CD14 exposure on the cell surface and priming microglia to stimulation by pro‐inflammatory factors. Loss of SORLA in gene‐targeted microglia impairs proper CD14 sorting and blunts pro‐inflammatory responses. Our studies indicate an important role for SORLA in shaping the inflammatory brain milieu, a biological process important to local immune responses in AD.

## Main Points

- SORLA directs CD14 to the cell surface, sensitizing microglia to inflammatory stimuli.
- SORLA activity in microglia may play an important role in shaping the inflammatory environment of the brain.

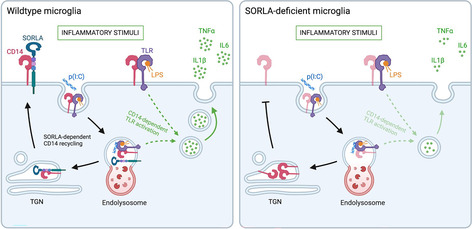

## 1 Introduction

Alzheimer's disease (AD) is the most common form of age‐related dementia affecting millions of patients worldwide. By now, genome‐wide association studies (GWAS) have been instrumental in identifying novel genes associated with the risk of onset and progression of this disease (Bellenguez et al. 2022; Kunkle et al. 2019; Lambert et al. 2013). Remarkably, many identified AD risk genes were linked to the activities of microglia (Hansen, Hanson, and Sheng 2018), supporting concepts of an important role for this immune cell type in AD pathology (Heneka, Kummer, and Latz 2014; Heppner, Ransohoff, and Becher 2015; Leng and Edison 2021). These observations are in line with emerging concepts that microglia exert crucial functions in the normal brain and influence the pathologic process of most diseases of the central nervous system (Wolf, Boddeke, and Kettenmann 2017).

The significance of some AD risk genes for microglia function is well established, as for the triggering receptor expressed on myeloid cells 2 (TREM2; Kleinberger et al. 2014) or CD33 (Griciuc et al. 2013). However, for other risk genes, a link to microglia activities remains poorly established. A prominent example is *SORL1*, the gene encoding the sortilin‐related receptor with A type repeats (SORLA) (reviewed in (Andersen, Rudolph, and Willnow 2016)). *SORL1* gene variants are associated with both familial (Holstege et al. 2017) as well as sporadic forms of AD (Bellenguez et al. 2022; Rogaeva et al. 2007). Damaging mutations in *SORL1* may affect as many as 2.75% of all familial AD patients (Holstege et al. 2022), while *SORL1* SNPs of genome‐wide significance represent some of the most protective genetic variants in AD known to date (Bellenguez et al. 2022). So far, a protective function for SORLA in the healthy aged brain has largely been attributed to its ability to act as a neuronal sorting receptor for the amyloid precursor protein (APP; Andersen et al. 2005) and for amyloid‐β peptides (Aβ; Caglayan et al. 2014), preventing built‐up of amyloidogenic products in the brain. Surprisingly, recent studies now suggest that noncoding risk SNPs in *SORL1* may not impact receptor expression in neurons, but in microglia, arguing for an important function of the receptor in this cell type in the context of AD (Nott et al. 2019). Yet, possible functions for SORLA in microglia received little attention so far, likely because robust expression in this cell type is a distinguishing feature of the human brain, not observed in experimental rodent models (Hansen, Hanson, and Sheng 2018).

Toward a better understanding of the physiological role of SORLA in human microglia, we performed functional analyses of induced pluripotent stem cell (iPSC)‐derived human microglia, wild‐type, or genetically deficient for this receptor. This approach has recently been established as a faithful strategy to generate a human cell type that shares many features with microglia of the human brain (Hasselmann and Blurton‐Jones 2020). Our analyses covered a wide spectrum of microglia activities, from cell migration, to phagocytosis, to immune responses, not to be constrained by prior hypothesis of receptor function in neurons. Our studies uncovered an important role for SORLA in sensitizing human microglia to pro‐inflammatory stimuli, a function linked to its ability to regulate cell‐surface sorting of CD14, a multifunctional pattern recognition receptor (PRR). Conceptually, impaired expression or activity of SORLA, seen with some risk SNP variants, may promote disease progression by impairing protective immune responses of microglia in the diseased brain.

## 2 Methods

### 2.1 Human Brain Specimens

**FIGURE 1:**
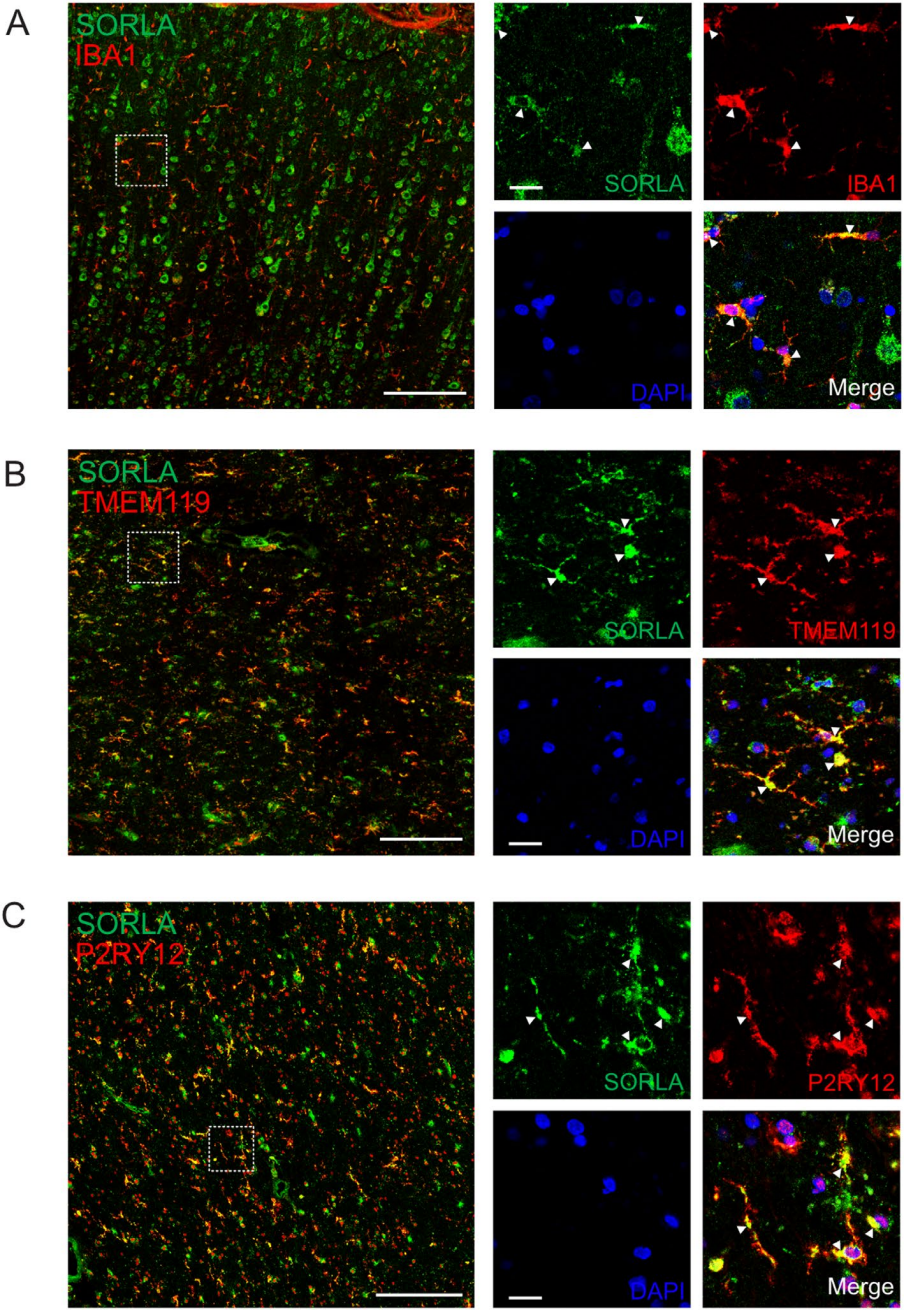
Expression of SORLA in microglia of the human brain. Immunohistochemical staining of SORLA (green) in human cortical brain tissue from two individual donors are shown. Sections from one donor were co‐stained for microglial marker IBA1 (red, A). Sections from a second donor were co‐stained for microglial marker TMEM119 (red, B) or P2RY12 (red, C). Nuclei were counterstained with DAPI (blue). White boxes in the overview images mark the higher magnification areas shown in the right panels. Arrowheads indicate SORLA immunoreactivity in IBA1^+^, TMEM119^+^, or P2RY12^+^ cells. Scale bars: 200 μm (overview images), 20 μm (magnified images).

Human brain sample used for Figure 1A constituted a left parietal cortex sample from a 75‐ to 80‐years‐old male patient obtained from Aarhus University Hospital undergoing surgery for a deep brain tumor (Louth et al. 2021). The sample was healthy brain tissue surgically excised to gain access to the tumor. Human brain samples used for Figure 1B,C constituted a 14‐h post‐mortem interval cortex autopsy tissue sample from a 75‐ to 80‐years‐old female AD patient (patient ID: BB000044) obtained from the Biobank of the Department of Neuropathology, Charité‐Universitätsmedizin Berlin (Radke et al. 2024). Detailed protocols for immunohistochemistry of the human brain samples are given in the Supporting Information Methods.

### 2.2 Generation of *SORL1* ‐Deficient iPSC Lines

The human‐induced pluripotent stem cell (iPSC) line HMGU001‐A/BIHi043‐A (https://hpscreg.eu/) was used as wild‐type control in this study. Isogenic *SORL1*‐deficient lines HMGUi001‐A‐18/BIHi043‐A (SORL1_KO Cl B1) and HMGUi001‐A‐19/BIHi043‐A (SORL1_KO Cl F2) were generated independently from the parental line by CRISPR/Cas9‐mediated genome editing, as described in Ludwik et al. 2023, using a gRNA targeting exon 1 in *SORL1* (5′‐CAGTAGCGTTCGCCCGAACA‐3′). An identical 8 bp frameshift deletion and predicted inactivation of *SORL1* in KO‐B1 and KO‐F2 was confirmed by PCR amplification and Sanger sequencing using primers 5′‐AGAAAGTGCGCGAAAGGGA‐3′ (forward) and 5′‐AAAACTGCTCACCTGTCCGT‐3′ (reverse). All iPSC lines were quality‐controlled by SNP‐karyotyping and confirmation of pluripotency, as described in Metzler et al. (2020), and routinely tested negative for mycoplasma. Details of the TaqMan Scorecard Assay (Applied Biosystems) to evaluate pluripotency are given in the Supporting Information, Methods.

### 2.3 Differentiation of iPSC Into Human Microglia

For maintenance, iPSC lines were cultured on Matrigel (Gibco, #356324) coated 6‐well plates in Essential 8 Flex Medium (Gibco #A2858501). The culture medium was changed every second day and the cells were passaged in clusters every 3–4 days at a density of 80% using 0.5 mM EDTA/PBS. The iPSC lines were maintained for 3–10 passages before starting a differentiation experiment. Differentiation of iPSC into microglia (iMG) was done using published protocols (McQuade et al. 2018).

In brief, iPSCs were differentiated into hematopoietic progenitors (HP) using the STEMdiff Hematopoietic Kit (Stem Cell Technologies, cat. #05310). To do so, iPSCs at a confluency of 70%–80% were passaged with ReLeSR (Stem Cell Technologies, cat. #05872) in E8 flex containing Matrigel‐coated 6‐well plates (day −1). Cell clusters of 100 cells were seeded at a density of 50–100 clusters per well. At Day 0, in wells with a total of 40–80 clusters, E8 flex medium was replaced with 2 mL of medium A. On Day 2, 1 mL of medium A was added to the well. On Day 3, the medium was replaced with 2 mL of medium B. At Days 5, 7, and 10, one milliliter of medium B was added. At Day 12, the media was gently resuspended in the wells using a 5 mL serological pipette to increase the yield of HPs. Collected HPs were centrifuged at 300 × *g* for 5 min and resuspended in microglia differentiation medium (DMEM/F‐12; 11039‐021, Gibco) containing 2× Insulin‐Transferrin‐Selenite (41400045, Thermo Fisher Scientific), 2× B27 (17504001, Thermo Fisher Scientific), 0.5× N2 (17502048, Thermo Fisher Scientific), 1× GlutaMAX (35050038, Thermo Fisher Scientific), 1× NEAA (11140035, Thermo Fisher Scientific), 400 μM monothioglycerol (M1753, Sigma), 5 μg/mL insulin (C‐52310, PromoCell), 100 ng/mL IL‐34 (200‐34, PeproTech), 50 ng/mL TGFβ1 (100‐21C, PeproTech), and 25 ng/mL M‐CSF (300‐25, PeproTech). Cells were seeded at a density of 200,000 cells in Matrigel‐coated 6‐well plates (Corning). On Days 14, 16, 18, 20, and 22, one milliliter of microglia differentiation medium was added to the well. At Day 24, five milliliter medium was transferred to a 15 mL falcon tube to spin down the cells at 300×g for 5 min. The supernatant was removed and the cell pellet was resuspended in 1 mL microglia differentiation medium and returned to the well. This procedure was repeated daily for Days 26 through 33. At Day 35, cell pellets were resuspended in microglia differentiation medium, containing 100 ng/mL CD200 (E‐PKSH032840, Elabscience) and 100 ng/mL CX3CL1 (300–31, PeproTech), for further maturation. On Day 37, one milliliter of microglia maturation medium was added to the well. Between Days 38 and 42, the microglia were used for functional studies as described in the following. Details on routine expression analyses using quantitative RT‐PCR and immunocytochemistry are given in the Supporting Information Methods.

### 2.4 Bulk RNA Sequencing

Six biological replicates of WT and SORLA KO‐B1 iMG from three independent differentiation experiments were used for bulk RNA sequencing analysis. On Day 38 of iMG differentiation, the cells were collected, centrifuged, and the cell pellets snap‐frozen. Total RNA from 500,000 iMG per sample was isolated using the RNeasy Mini kit (Qiagen, #74104), with DNase treatment (15 min, RT) according to manufacturer's instructions. RNA concentration was measured with Qubit fluorometer and RNA integrity was assessed with TapeStation. 500 ng of RNA per sample was used to create RNA‐seq libraries using the Illumina TruSeqTM Stranded Total RNA Library Prep Gold (Illumina, #20020598). Libraries were sequenced on the Illumina NovaSeq 6000 platform in paired‐end 100 nt mode, aiming at 50 million reads per sample. RNA‐sequencing data were processed and interpreted by Omiics (Aarhus, Denmark; https://omiics.com/), using in‐house bioinformatic analysis pipelines. RNA‐sequencing read integrity was verified using FastQC (0.12.1), Picard (2.3.1), and Multiqc (1.9). Trim Galore (0.6.10) was used to trim adapters and filter poor quality reads. Remaining reads were mapped to the human genome (hg19) using STAR (2.7.11b) and gene expression was quantified using featureCounts (2.0.0) with gene annotations from Gencode release 37. Differential expression analysis was performed using DESeq2 (1.40.2) in R by applying 0.05 FDR cutoff. A batch correction was added to DESeq2 setup to correct for batch differences between the three experiments, as observed in the PCA plot. Gene Ontology (GO) analysis was done using the clusterProfiler (4.8.2) R package. Volcano plots were generated using the ggplot2 (3.4.4) R package.

### 2.5 Functional Analysis of Human Microglia

Functional analyses of induced human microglia (iMG) using (i) scratch wound assay, (ii) phagocytosis assays, or (iii) pro‐inflammatory response by cytokine profiling are detailed in the Supporting Information Methods.

### 2.6 Flow Cytometry

For flow cytometry, iMG were harvested and resuspended in incubator‐equilibrated microglia maturation media as a single‐cell suspension at a concentration of 2 × 10^5^ cell/mL. 250 μL cell suspension was transferred to Eppendorf tubes (50,000 iMG/tube) and incubated with 100 ng/mL LPS or PBS (unstimulated control) for 20 h in the incubator before the immunostaining. For immunostaining, an antibody mastermix was used that consisted of 2 μL anti‐CD14‐PE (12‐0149‐42, Thermo Fisher Scientific), 1 μL LIVE/DEAD Fixable Violet Dead Cell (L34955; Invitrogen), as well as 47 μL flow media (1% BSA, 0.5 mM EDTA in PBS). Blank and single stains were used to set the positive boundaries for fluorescence intensity analysis. Fifty microliters of mastermix was added to each sample, followed by incubation for 30 min at 4°C. Next, 400 μL flow medium was added to stop staining. Samples were centrifuged at 300 × *g* for 5 min and washed twice in flow media before FLOW cytometry analysis using a BD FACSymphony A3 Cell Analyzer (BD Biosciences). FLOW cytometry data were analyzed using FlowJo version 10 and presented as mean fluorescence intensity (MFI).

For imaging flow cytometry, iMG were harvested on the day of the experiment and resuspended in microglia differentiation media as single cell suspension at a concentration of 1 × 10^6^ cell/mL, and 100 μL of cell suspension each was added to Eppendorf tubes (100,000 cells/tube). Blocking with purified human IgG (100 μg/mL, Beriglobin, CSL Behring) was used to prevent nonspecific antibody binding (Andersen et al. 2016). Samples were then washed in 1 mL 0.5% BSA in PBS, centrifuged for 5 min at 1000 × *g*, and the supernatant discarded down to 100 μL. One microliter of LIVE/DEAD Fixable Near IR (780) (L34994; Invitrogen) and 5 μL LysoTracker Red DND‐99 (1:100, L7528; Invitrogen) were added and samples were stained at 4°C for 30 min. Blank and single stains were used to set the positive boundaries for fluorescence intensity analysis. Next, samples were washed in 1 mL 0.5% BSA in PBS, centrifuged for 5 min at 1000 × *g*, and the supernatant was discarded to yield a 30 μL volume. Then, samples were fixed in 100 μL 4% PFA for 20 min at 4°C. Samples were washed in 1 mL 0.5% BSA in PBS, centrifuged for 5 min at 1000 × *g*, and the supernatant was discarded to yield a 100 μL volume. Then, 1 mL PBS, containing 0.1% Saponin and 0.5% BSA, was added and samples were incubated for 10 min at 4°C for permeabilization. Following, samples were centrifuged for 5 min at 1000 × *g* and the supernatant was discarded to yield a 100 μL volume. Finally, samples were stained with 10 μL FITC‐labeled mouse anti‐human CD14 (Clone M5E2, 555397, BD) for 30 min at 4°C, washed in 1 mL PBS (0.1% Saponin, 0.5% BSA), and centrifuged for 5 min at 1000 × *g*. The supernatants were discarded to yield a 50 μL volume and samples were kept on ice until analysis. Imaging flow cytometry was performed using an Amnis ImageStreamX MKII (Amnis, Seattle, WA) with sensitivity set to high and a 60× image magnification. Images from 5000 to 10,000 cells were recorded for all samples. Data were analyzed using the IDEAS software package (Amnis). The intracellular cell compartment was determined from the brightfield image, using the morphology erode 3 mask in IDEAS. Co‐localization of CD14 and lysosomal marker was determined from the combination of bright detail spots and similarity features for channels 2 and 3, respectively. Co‐localization was established for feature values above 2 Arbitrary Units (A.U).

### 2.7 Statistical Analyses

The number *n* represents biological replicates collected from a minimum of three independent differentiation experiments. For live cell imaging experiments of motility and phagocytosis, the *n* represents individual experiments. For co‐localization studies, *n* is the number of cells analyzed from two independent experiments. Statistical analyses were conducted by ANOVA or Mixed‐effect model and corrected for multiple testing using Dunnett in GraphPad Prism version 10, unless stated otherwise. Data are presented as mean ± standard error of the mean (SEM). The level of statistical significance is reported as *p < 0.05, **p < 0.01, ***p < 0.001, ****p < 0.0001, or as nonsignificant (ns). Further details of statistical analyses are specified in the respective figure legends.

## 3 Results

### 3.1 SORLA Is Expressed in Human Microglia In Vivo and in Culture

Prior single‐cell sequencing studies have suggested that microglia is the cell type in the human brain with the highest levels of *SORL1* transcript (Gosselin et al. 2017; Hansen, Hanson, and Sheng 2018). However, expression of the receptor in microglia of the human brain has not been validated at the protein level yet. Performing immunohistochemical analyses for SORLA on human cortical brain sections from two independent donors, we now document colocalization of the receptor with the microglia markers ionized calcium‐binding adapter molecule (IBA1; Figure 1A), transmembrane protein 119 (TMEM119; Figure 1B), and purinergic receptor P2Y12 (P2RY12; Figure 1C), confirming receptor expression in human microglia in vivo.

**FIGURE 2:**
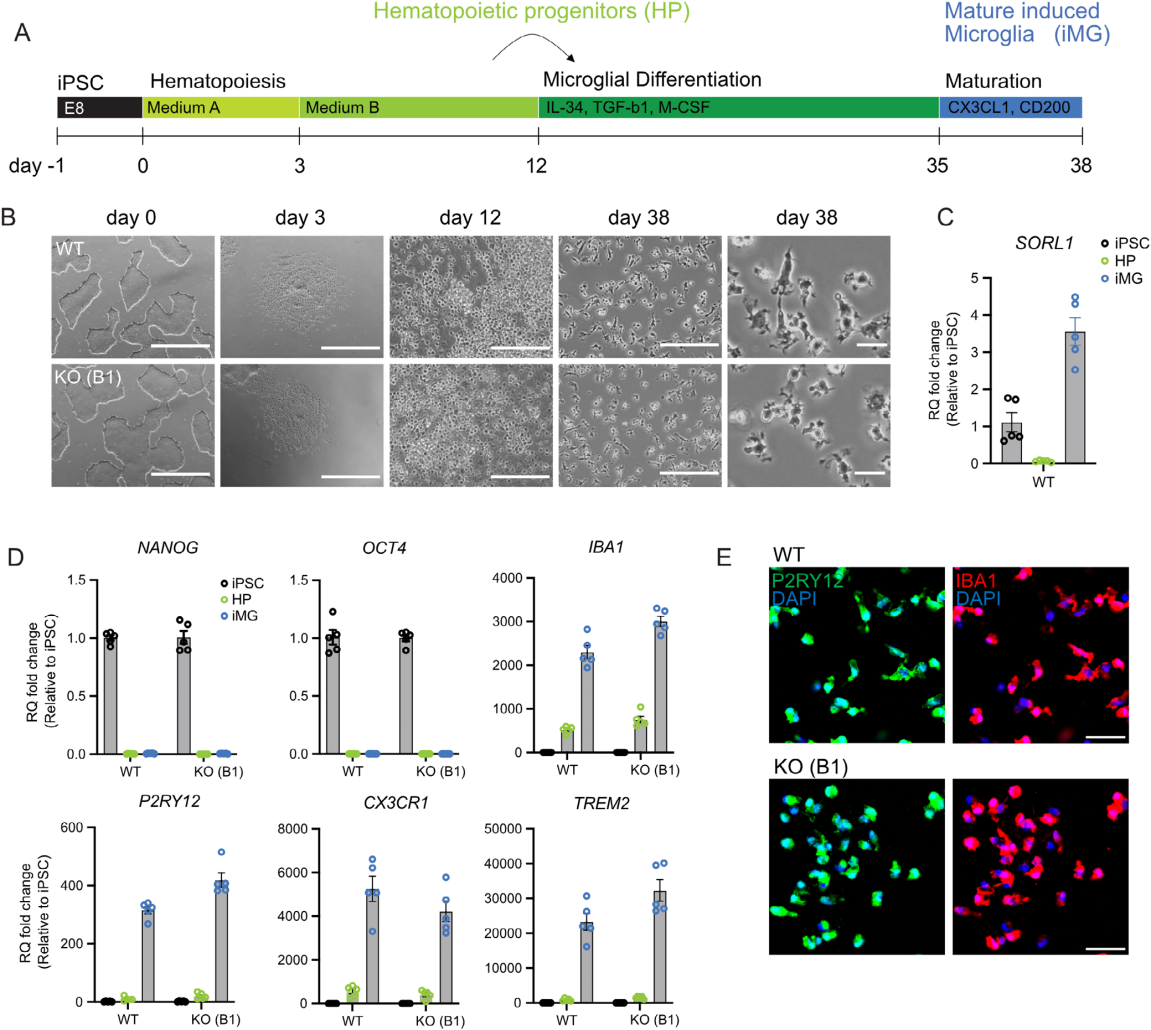
Differentiation of WT and KO‐B1 iPSCs into human microglia. (A) Protocol used for differentiation of iPSCs into microglia (iMG) (for details, see Methods). (B) Phase contrast images of iPSCs, hematopoietic progenitors (HP), and iMG at different stages of microglia differentiation of WT and KO‐B1. For Day 38 of differentiation, overview as well as higher magnification images are given. Scale bars: 1000 μm (Day 0 and Day 3), 200 μm (Day 12 and Day 38), 20 μm (Day 38 zoom‐in). (C) Quantitative RT‐PCR of *SORL1* transcript levels in WT iPSCs (Day 0), HP (Day 12), and iMG (Day 38). Relative quantification (RQ) fold changes represent 2^−ddCt^ relative to iPSC. *GAPDH* and *TBP* were used as reference genes (*n* = 5 biological replicates). (D) Quantitative RT‐PCR of pluripotency markers *NANOG*, *OCT4* as well as microglia markers *IBA1*, *P2RY12*, *CX3CR1*, and *TREM2* in WT and KO‐B1 iPSCs (Day 0), HPs (Day 12), and iMG (Day 38). Relative quantification (RQ) fold changes represent 2^−ddCt^ relative to iPSC. *GAPDH* and *TBP* were used as reference genes (WT *n* = 5, KO‐B1 *n* = 5 biological replicates of all conditions). (E) Immunofluorescence detection of IBA1 (red) and P2RY12 (green) demonstrates comparable homogeneity of iMG cell preparations from WT and SORLA KO‐B1 iPSCs. Nuclei were counterstained with DAPI (blue). Scale bars: 50 μm.

To explore the functional significance of SORLA in human microglia, we generated isogenic human induced pluripotent stem cell (iPSC) lines, either wildtype (WT) or genetically deficient for *SORL1* (KO) (Figure S1A). Successful ablation of SORLA expression in two independent KO iPSC lines (KO‐B1 and KO‐F2) was confirmed by Sanger sequencing (Figure S1B) and Western blot analysis (Figure S1C). SORLA deficiency did not impact the expression of the related receptor sortilin (Figure S1C), nor iPSC morphology or growth (Figure S1D). Also, pluripotency, as tested by expression of pluripotency markers *SOX2*, *OCT4*, and *NANOG* (Figure S1E), or the ability to generate all three germ layers (Figure S1F–H) was unaffected. WT and KO iPSCs were differentiated into induced microglia (iMG) using established protocols (McQuade et al. 2018) (Figure 2A). The differentiation protocol produced cells with ramified microglia‐like morphology, comparable in both *SORL1* genotypes (Figure 2B), with pronounced *SORL1* transcript levels in WT iMG (WT, Figure 2C). Assessment of differentiation of WT and KO cells at Day 0 (iPSC), Day 12 (hematopoietic progenitor cells), and Day 38 (iMG) showed the expected reduction in expression of pluripotency markers *NANOG* and *OCT4*, and a concomitant increase in expression of microglia markers *IBA1*, *P2RY12*, *CX3CR1*, and *TREM2* (Figure 2D), with no discernable differences between *SORL1* genotypes as exemplified for clone KO‐B1. Also, immunostainings confirmed that the majority of iMG from both genotypes expressed P2RY12 and IBA1 (Figure 2E). Taken together, our differentiation protocol generated a homogeneous population of microglia‐like cells comparable between WT and KO genotypes, documenting the applicability of our cell model to study SORLA functions in human microglia.

### 3.2 SORLA Deficiency Induces Transcriptome Changes in Microglia Associated With Intracellular Vesicle Biology and Immune Cell Activation

**FIGURE 3:**
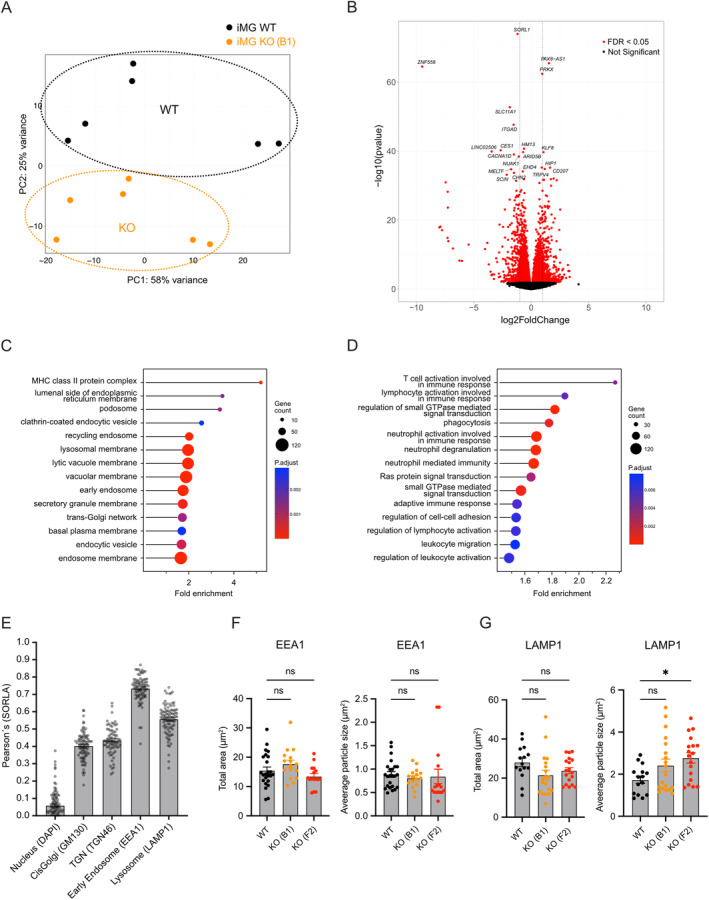
SORLA deficiency in iMG induces transcriptome changes associated with intracellular vesicle biology and immune cell activation. (A) Principal component analysis of bulk RNA sequencing data from WT and KO‐B1 iMG, documents separation of samples into the two genotype groups (*n* = 6 biological replicates). (B) Volcano plot based on comparative bulk RNAseq showing fold change of differential gene expression in WT versus KO‐B1 iMG as red dots (adjusted *p*‐value < 0.05). The 20 most significantly expressed genes are listed in the volcano plot. (C, D) Gene ontology (GO) analyses of differentially expressed genes (DEGs) in WT versus KO‐B1 iMG performed using R. (C) Cellular components enriched in KO iMG are associated with vesicular trafficking in endo‐lysosomal compartments. (D) Biological processes enriched in KO iMG are linked to immune cell activation, phagocytosis, and cell migration. Fold enrichment is calculated by dividing the percentage of DEGs in the respective GO term by the corresponding percentage in the background gene list. Size of the dots represents the number of DEGs in the respective GO term. Color (blue‐to‐red) indicates the level of significance, by adjusted *p*‐value, of the respective GO term. (E) Localization of SORLA to the indicated subcellular compartments in WT iMG. The extent of localization was determined by Pearson's correlation coefficient of immunostainings exemplified in Figure S2 (*n* = 60–100 cells per condition). (F, G) Total cell area (left panel) and average vesicle size (right panel) of endosomal (F) and lysosomal compartments (G) in WT as compared to KO‐B1 and KO‐F2 iMG lines. Data were obtained by ImageJ particle analysis of immunostainings for EEA1 (F) and LAMP1 (G) exemplified in Figure S3. (*n* = 15–24 cells per marker). Statistical significance of data was determined using one‐way ANOVA corrected for multiple testing by Dunnett.

To identify so far unknown functions for SORLA in human microglia, we performed comparative bulk RNA sequencing comparing the transcriptomes of WT and KO‐B1 iMG. These experiments showed a clear separation of the two genotypes by principal component analysis (PCA; Figure 3A). Among the differentially expressed genes (DEGs), *SORL1* was the most significantly downregulated gene in KO‐B1 iMG (Figure 3B). Another top downregulated gene was integrin subunit alpha D (ITGAD). ITGAD shares close structural similarity with β2 integrin family members ITGAM and ITGAX, highly expressed in microglia (Juul‐Madsen et al. 2020) and associated with increased inflammation in AD (Juul‐Madsen et al. 2024; Wilton et al. 2023; Yoo et al. 2024).

Gene Ontology (GO) analysis of DEGs revealed links to cellular organelles known to harbor SORLA in other cell types, including endosomes, lysosomes, and the *trans*‐Golgi network (TGN; Figure 3C; Caglayan et al. 2014; Dumanis et al. 2015). Interestingly, GO terms of biological processes were also enriched for central immune cell functions, such as immune cell reactivity, antigen presentation, leukocyte migration, and phagocytosis (Figure 3D), suggesting the involvement of SORLA in immunomodulatory functions of microglia.

To validate the findings from the GO analyses, we tested the subcellular localization of SORLA in iMG using immunocytochemistry. Our data showed a robust expression of SORLA in WT but not in KO iMG (Figures S2 and S3A,B). Co‐immunostaining with markers of various subcellular compartments revealed predominant co‐localization of SORLA with the early endosomal marker EEA1, as well as moderate co‐localization with the lysosomal marker LAMP1 (Figures 3E and S2). No differences in the morphological appearance of early endosomes were seen when comparing WT with KO‐B1 or KO‐F2 iMG for total endosomal area or vesicle size (Figures 3F and S3A). For lysosomes, we noted a subtle increase in vesicle size, but not in total lysosome area per cell (Figures 3G and S3C). However, this trend only reached significance in iMG from KO‐F2, but not from KO‐B1.

### 3.3 SORLA Expression Does Not Impact Motility or Phagocytic Properties of Human iMG

To further characterize biological processes enriched in our GO analyses, we performed in‐depth investigations focusing on cell motility, phagocytosis, and immune cell activation, functions central to the role of microglia in AD pathology.

**FIGURE 4:**
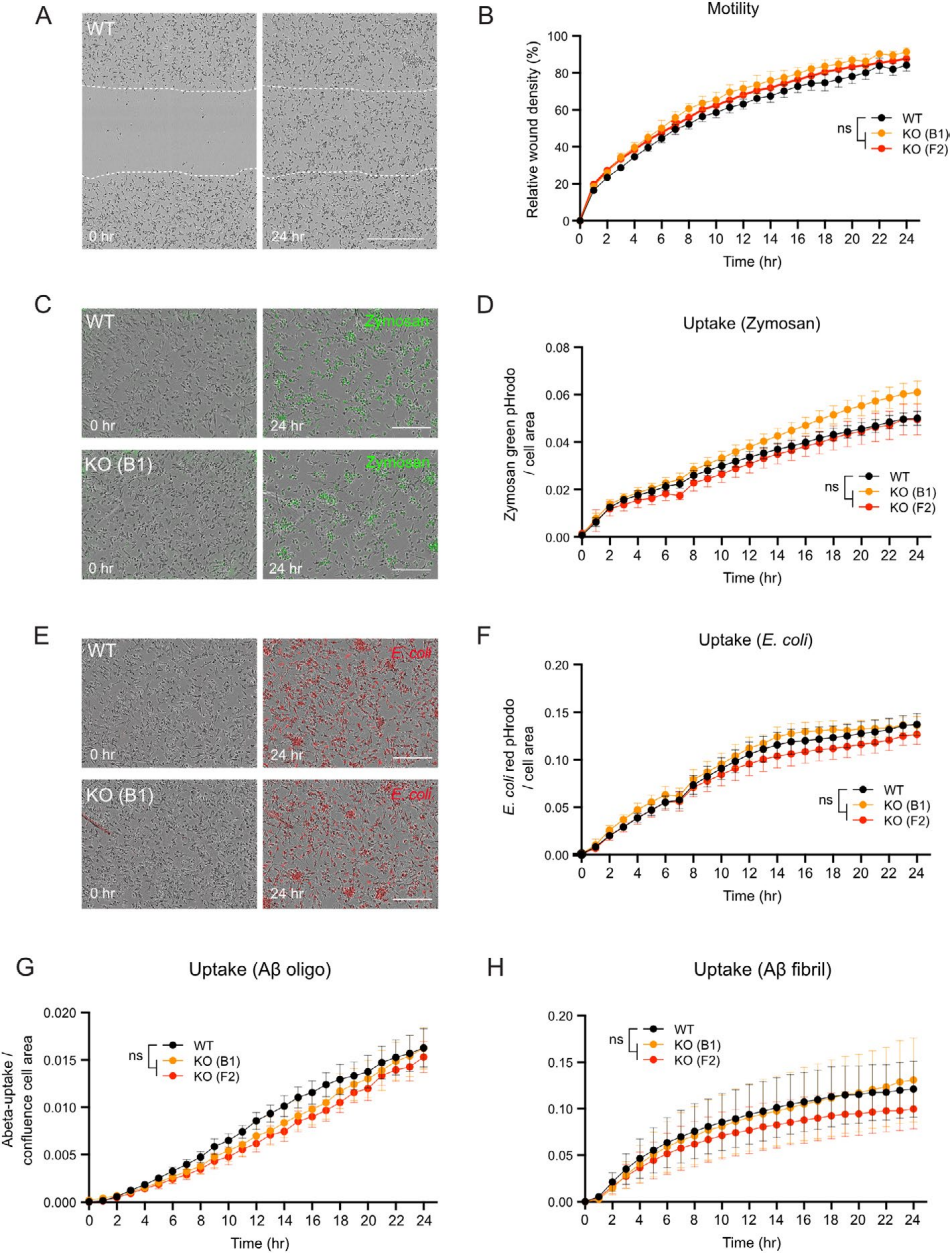
SORLA deficiency does not impact motility or phagocytic properties of human iMG. (A, B) Motility of WT and KO (B1 and F2 clones) iMG was tested using the scratch‐wound assay. Phase contrast images of WT iMG at 0‐ and 24‐h post‐scratch are given in A. White lines mark the scratch area. Scale bar: 500 μm. Quantifications of relative wound densities in WT and KO iMG cell layers based on cell confluency in scratched versus nonwounded areas at the indicated time points post‐scratch are given in B (WT *n* = 4, KO‐B1 *n* = 3, KO‐F2 *n* = 4 independent experiments). (C, D) Phagocytosis of Zymosan Green pHrodo particles by WT and KO‐B1 and KO‐F2 iMG as analyzed by live fluorescence imaging using Incucyte SX5. Phase contrast images of cells documenting Zymosan uptake (green fluorescence signal) at 0 and 24 h after particle addition to WT and KO iMGs are shown in C. Scale bar: 200 μm. Quantification of particle uptake as ratio of Zymosan signal normalized to cell area over time are shown in D (WT *n* = 5, KO‐B1 *n* = 5, KO‐F2 *n* = 3 independent experiments). (E, F) Phagocytosis of *E*. *coli* Red pHrodo particles in WT and KO‐B1 and KO‐F2 iMG as analyzed by live fluorescence imaging using Incucyte SX5. Phase contrast images of cells highlighting *E*. *coli* uptake (red fluorescence signal) at 0 and 24 h after particle addition are shown in E. Scale bar: 200 μm. Quantification of *E*. *coli* Red pHrodo particle uptake in WT and KO iMG over time, determined as the ratio of *E*. *coli* red signal normalized to cell area, are given in F (WT *n* = 3, KO‐B1 *n* = 3, KO‐F2 *n* = 3 independent experiments). (G, H) Phagocytosis of oligomeric (G) or fibrillary (H) forms of Fluor 488‐labeled amyloid‐β particles (HiLyte Aβ) in WT and KO iMG. Uptake was quantified as cellular fluorescence of HiLyte Aβ normalized to the cell area. (WT *n* = 4, KO‐B1 *n* = 4, KO‐F2 *n* = 4 independent experiments). Statistical significance of data was determined by two‐way ANOVA with repeated measures (G, H) or by mixed‐effect model (B, D, F).

SORLA has previously been associated with smooth muscle cell migration in the vessel wall (McCarthy et al. 2010; Zhu et al. 2002). To query a similar role for the receptor in the migration of microglia, we analyzed basal motility of WT and KO iMG using a scratch wound assay, combined with live cell imaging. In these studies, KO‐B1 and KO‐F2 iMG showed comparable abilities to WT to migrate and re‐populate the wounded space within 24 h (Figure 4A,B). Phagocytosis to remove excess synaptic material and noxious substances is another key function of microglia in the brain. Therefore, we assessed the phagocytic properties of WT and KO iMG by measuring the uptake of fluorescence‐labeled particles of different origins using live cell imaging. In these experiments, Zymosan and *Escherichia coli* pHrodo‐labeled particles were phagocytosed to the same extent in WT and both KO iMG lines (Figure 4C–F).

With relevance to AD, microglia‐mediated uptake of Aβ aggregates is a major defense mechanism to protect the brain from amyloid‐induced pathology (Hickman, Allison, and El Khoury 2008). We have previously shown that SORLA acts as an intracellular sorting receptor for soluble Aβ, directing newly produced amyloid peptides to lysosomes for catabolism (Caglayan et al. 2014). To test whether SORLA may also contribute to the clearance of extracellular amyloid by microglia, we assessed the phagocytosis of fluorescently labeled Aβ peptides in our iMG lines. In these studies, oligomeric or fibrillary forms of Aβ were cleared with comparable rates in WT and both KO iMG lines (Figure 4G,H). Taken together, our studies failed to document a prominent role for SORLA in migration or phagocytic activity of human microglia.

### 3.4 SORLA Deficiency Impairs the Pro‐Inflammatory Response of Human iMG

To elucidate hitherto unknown functions for SORLA unique to microglia cell biology, we focused on further investigations on receptor functions related to immune cell activation, suggested by our GO analyses.

**FIGURE 5:**
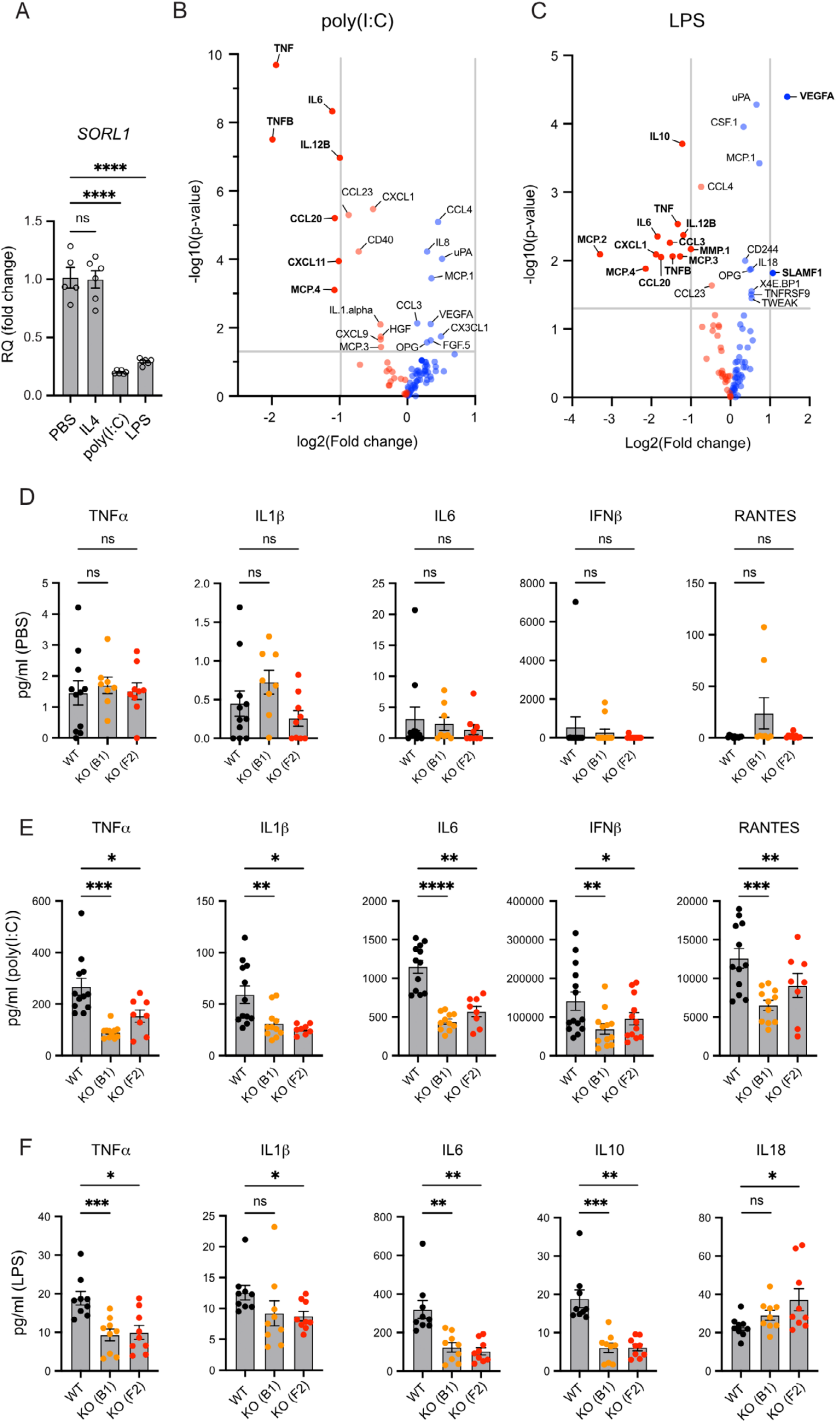
SORLA deficiency impairs the pro‐inflammatory response of human iMG. (A) Quantification of *SORL1* transcript levels in WT iMG stimulated with IL4 (anti‐inflammatory), LPS (pro‐inflammatory), or poly(I:C) (pro‐inflammatory). Relative quantification (RQ) fold changes represent 2^−ddCt^ relative to unstimulated control (PBS). *GAPDH*, *TBP*, and *HPRT1* were used as reference genes (PBS *n* = 5, IL4 *n* = 6, poly(I:C) *n* = 6, LPS *n* = 6 biological replicates). (B, C) Multiplex immunoassays of 92 inflammatory biomarkers (Olink inflammation panel) were performed on media samples from WT and KO‐B1 iMG treated with 10 μg/mL poly(I:C) (B), or 100 ng/mL LPS (C) for 24 h. Differential expression levels of tested markers are given as Volcano plots with log2(fold change) and −log10(*p*‐value). Red and blue dots indicate molecules that are downregulated or upregulated in KO media samples, respectively. Gray horizontal and vertical lines represent nonadjusted *p* values equal to 0.05 and log2(fold change) of −1 and 1, corresponding to a halving or doubling in protein expression, respectively. Proteins with a *p*‐value < 0.05 are listed in the plot. Proteins with *p*‐value < 0.05 and log2(fold change) less than −1 and above 1 are highlighted and labeled in bold font. (D, E) Multiplex ELISA of TNFα, IL1β, IL6, IFNβ, and RANTES levels in media samples from unstimulated control (PBS) (D) or poly(I:C) (E) treated WT as well as KO‐B1 and KO‐F2 iMG (*n* = 8–12 biological replicates for all conditions and genotypes). (F) Multiplex ELISA of TNFα, IL1β, IL6, IL10, and IL18 levels in media samples from WT as well as KO‐B1 and KO‐F2 iMG treated with LPS (*n* = 9 for all conditions and genotypes). Statistical significance of data was determined using one‐way ANOVA (A) with repeated measures (F) or mixed‐effect model (D, E) and corrected for multiple testing by Dunnett.

To establish the relevance of SORLA for immune cell activation, we first analyzed *SORL1* transcript levels in iMG treated with triggers of inflammatory processes, namely polyinosinic–polycytidylic acid (poly(I:C)) and lipopolysaccharide (LPS), or with the anti‐inflammatory cytokine interleukin‐4 (IL4). We found that *SORL1* transcript levels strongly decreased following treatment with poly(I:C) and LPS, but did not change in response to IL4 (Figure 5A), linking SORLA with pro‐inflammatory activation of microglia. To explore this hypothesis further, we performed a multiplex immunoassay screen for 92 inflammatory markers in media samples from WT and KO‐B1 iMG stimulated with poly(I:C) or LPS. Both genotypes showed a massive increase in major pro‐inflammatory cytokines, including TNFα and IL6, when compared to the unstimulated genotype controls (Figure S4). However, when comparing inflammatory profiles using a ± 1 log fold change and an FDR < 0.05 cut‐off, 7 out of 92 (poly(I:C); Figure 5B) and 12 out of 92 (LPS; Figure 5C) markers were decreased in KO as compared to WT iMG. By contrast, none of 92 (poly(I:C); Figure 5B) or 2 out of 92 (LPS; Figure 5C) factors were increased in KO iMG. Markers with decreased levels in stimulated KO iMG common to poly(I:C) and LPS treatment conditions included central pro‐inflammatory molecules TNFα, TNFβ, IL6, and CCL20 (Figure 5B,C).

To validate the findings of an impaired pro‐inflammatory response in microglia lacking SORLA, we compared the concentrations of selected pro‐inflammatory cytokines in media samples from WT as well as KO‐B1 and KO‐F2 iMG using targeted ELISA. No differences in tested marker levels were seen comparing unstimulated WT and KO iMG (Figure 5D). By contrast, for the poly(I:C)‐treated condition, we confirmed a robust reduction in levels of TNFα, IL1β, and IL6 in supernatants from both KO iMG lines (Figure 5E). Furthermore, we identified a strong reduction for poly(I:C)‐associated endo‐lysosome response factors IFNβ and RANTES (Figure 5E), which were not included in the multiplex library. For the LPS‐treated condition, targeted ELISA confirmed reduced levels of TNFα, IL1β, and IL6, as well as a pronounced reduction in IL10 and a concomitant increase in IL18 in supernatant from both KO clones when compared to WT (Figure 5F). Jointly, these results demonstrated a reduced response of SORLA‐deficient iMG to various pro‐inflammatory stimuli, arguing for a generalized role of the receptor in facilitating inflammatory activation of human microglia.

### 3.5 SORLA Is a Sorting Receptor That Determines Surface Levels CD14 in Human iMG

To elucidate possible molecular mechanisms whereby SORLA may promote pro‐inflammatory activation of microglia, we focused on CD14, a multifunctional pattern recognition co‐receptor involved in both LPS (Wright et al. 1990) and poly(I:C) (Lee et al. 2006) signaling. Trafficking of CD14 between cell surface and endo‐lysosomal compartments is central to its pro‐inflammatory action (Ciesielska, Matyjek, and Kwiatkowska 2021). At the cell surface, CD14 acts as co‐receptor to Toll‐like receptor TLR4, facilitating the binding of LPS that generates a Myd88‐dependent pro‐inflammatory response through the release of cytokines, such as TNFα (Jiang et al. 2005). In addition, CD14 governs LPS‐induced internalization and trafficking of TLR4 to endo‐lysosomal compartments, essential for signaling and release of IFNβ and RANTES (Zanoni et al. 2011). CD14 is also involved in pro‐inflammatory signaling through poly(I:C) by delivering it to endosomes to engage TLR3, resulting in the release of IFNβ and RANTES (Baumann et al. 2010; Lee et al. 2006). Finally, at the cell surface, membrane‐bound CD14 may be subject to proteolytic cleavage, releasing a soluble fragment (sCD14) that shares functions with the membrane receptor in terms of pathogen response and inflammatory modulation (Viriyakosol et al. 2000). Ultimately, our choice of testing CD14 as a molecular target of SORLA action was based on its established role as trafficking receptor, sorting proteins between cell surface, TGN, and endo‐lysosomal compartments.

**FIGURE 6:**
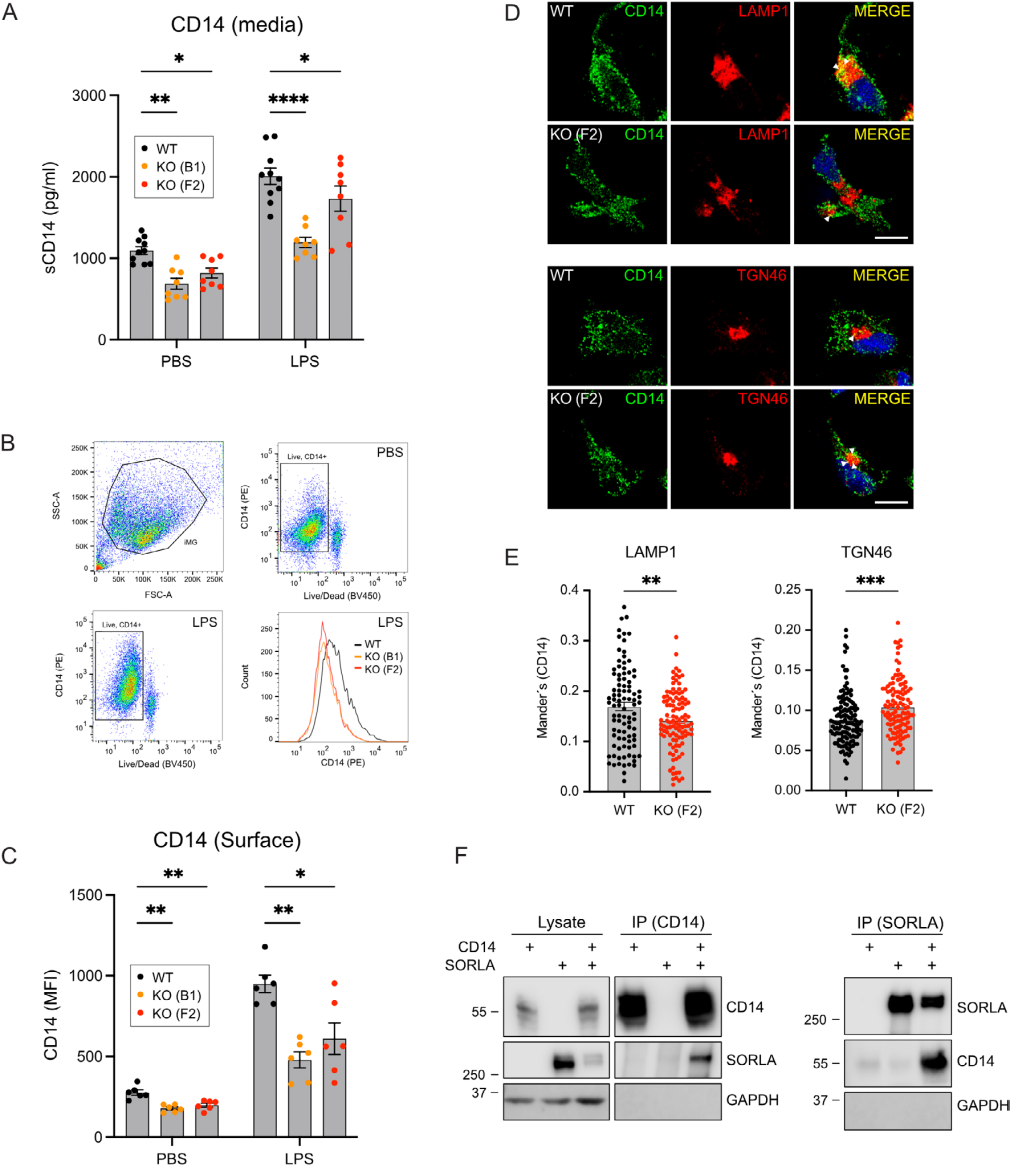
SORLA is a sorting receptor for CD14 in human iMG. (A) ELISA of soluble CD14 (sCD14) levels in media samples from PBS or LPS stimulated WT, KO‐B1, and KO‐F2 iMG (*n* = 8–10 biological replicates for all conditions and genotypes). (B, C) Flow cytometry‐based analysis of CD14 expression at the cell surface of PBS or LPS stimulated WT, KO‐B1, and KO‐F2 iMG. In panel B, gating of cells for the analysis by forward‐side scatter (upper left panel) followed by live–dead (BV450) and CD14 (PE) stains are shown. Panel C depicts quantification of CD14 signals in the indicated iMG lines as analyzed by mean fluorescence intensity (*n* = 6 biological replicates for all conditions and genotypes). (D) Immunofluorescence detection of CD14 (green) and markers of subcellular compartments in WT and KO‐F2 iMG. White arrowheads exemplify colocalization of CD14 with LAMP1 or TGN46 in the respective merged images. Scale bars: 10 μm. (E) Analysis of CD14 localization to LAMP1^+^ or TGN46^+^ cell areas determined by Mander's correlation coefficient of immunostainings exemplified in D (*n* = 100 cells per condition from two independent experiments). (F) Co‐immunoprecipitation of SORLA and CD14 from transfected HEK293 cells. Immunoprecipitation of CD14 (IP (CD14)) co‐precipitates SORLA in co‐transfected cells, but not in cells transfected with CD14 or SORLA expression constructs only (left panel). Immunoprecipitation of SORLA (IP (SORLA)) co‐precipitates CD14 in co‐transfected cells (right panel). GAPDH was not co‐precipitated in any of the experiments. The migration of protein marker bands of the indicated molecular weights (in kDa) are given. Statistical significance of data was determined using Student's *t*‐test (E), two‐way ANOVA with repeated measures (C), or the mixed‐effect model (A) and corrected for multiple testing by Dunnett.

Levels of sCD14 were significantly reduced in cell media from unstimulated KO‐B1 and KO‐F2 iMG compared to WT cells, as documented by ELISA (Figure 6A), arguing for reduced sorting of CD14 to the cell surface in mutant cells. Reduced levels of sCD14 in media from KO iMG were maintained following LPS stimulation (Figure 6A), suggesting that impaired cell surface sorting of CD14 precedes pro‐inflammatory activation. In line with this assumption, levels of CD14 present at the cell surface in both unstimulated and LPS‐stimulated conditions were reduced in KO‐B1 and KO‐F2 compared with WT iMG, as shown by flow cytometry (Figure 6B,C). Co‐immunostaining of CD14 with markers of various intracellular compartments documented a relative increase in immunoreactivity in the TGN, and a concomitant decrease in lysosomes, in SORLA‐deficient iMG versus WT (Figure 6D,E). Finally, evidence for a direct role of SORLA in intracellular sorting of CD14 was supported by co‐immunoprecipitation experiments in transfected HEK293 cells. In these studies, immunoprecipitation of SORLA co‐precipitated CD14, and vice versa (Figure 6F).

**FIGURE 7:**
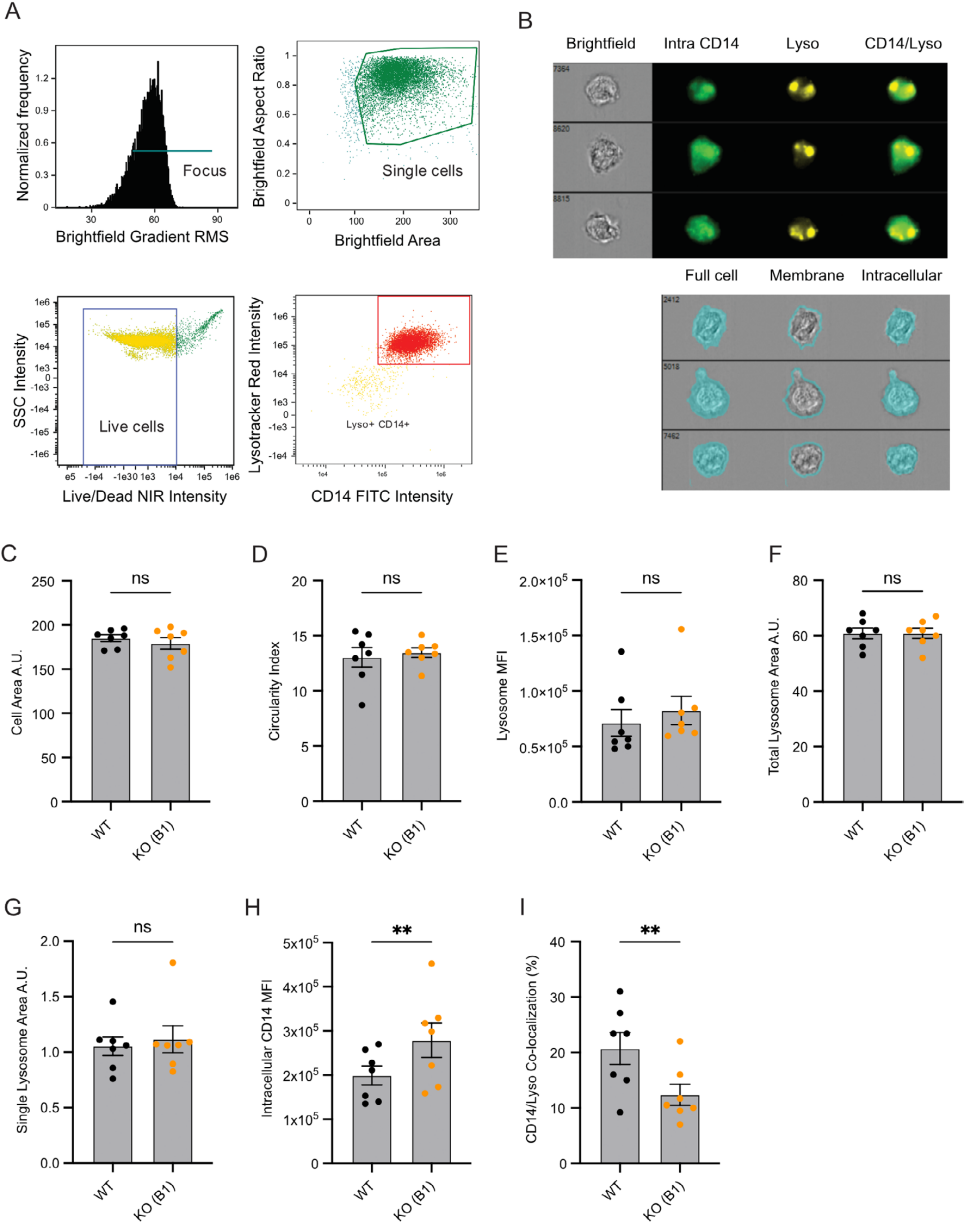
SORLA regulates the intracellular distribution of CD14 in iMG. Intracellular distribution of CD14 in WT and KO‐B1 iMG was analyzed by imaging flow cytometry (*n* = 7 biological replicates from five independent experiments, each replicate represents an average of 5000–10,000 cells tested). (A) Gating of cell populations for analysis by focus (upper left), single cells (upper right), viability (lower left), or CD14/Lysotracker positive cells (lower right panel) are shown. (B) Brightfield images as well as images of immunosignals for intracellular CD14, Lysotracker, as well as CD14 and Lysotracker overlays in three representative WT cells. Full cell masks generated from the brightfield images were used to separate membrane and intracellular compartments. The cell membrane compartment was defined as the three outmost pixels (300 × 300 nm/pixel) of the full cell mask around the entire circumference of the cell as indicated by teal coloring. The intracellular compartment includes the total mask with the subtraction of the membrane mask. (C, D) Quantification of cell size (C; in arbitrary units, A.U.) and shape (D; circularity index) in WT and KO‐B1 iMG based on the full cell mask. (E–G) Quantification of intracellular Lysotracker signal was used to determine total mean fluorescence intensity of lysosomes (MFI; E), total lysosomal area (F), as well as single lysosome vesicle size (G). (H) Quantification of intracellular CD14 signal, measured by MFI. (I) Quantification of co‐localization between intracellular CD14 and Lysotracker, analyzed as percentage of CD14 signal located in Lysotracker positive areas. Statistical significance of data was determined by paired Student's *t*‐test.

To corroborate a suspected role for SORLA in cell‐surface sorting of CD14 in a second independent assay, we performed quantitative single‐cell fluorescence microscopy using imaging flow cytometry to study the intracellular distribution of immunostained CD14 in WT and KO‐B1 iMG. Cells were gated for imaging focus, single cells, viability, as well as Lysotracker and CD14 signals (Figure 7A). In Figure 7B, representative images of cells with various morphologies included in the analyses illustrate how membrane and intracellular compartments of the cells were defined (see figure legend for details). When analyzed for cell size and shape (i.e., circularity), no differences were seen comparing genotypes (Figure 7C,D). The same observation was made for KO‐F2 iMG (Figure S5A,B).

To assess the extent of CD14 localization to endo‐lysosomal compartments, we quantified the intracellular fluorescence signals from immunostaining of CD14 and from detection of lysosomes using Lysotracker in WT versus KO‐B1 or KO‐F2 iMG. Total lysosomal signal (Figure 7E and S5C), total lysosome area (Figure 7F and S5D), as well lysosome vesicle size (Figure 7G and S5E) all were comparable in wildtype and receptor‐deficient iMG, indicating largely unimpaired lysosomal biogenesis in SORLA‐deficient iMG. However, we detected a distinct increase in intracellular CD14 signal in both KO iMG lines as compared to WT cells (Figure 7H and S5F), corroborating a defect in cell surface sorting in the absence of SORLA. Confirming data from co‐immunostainings in Figure 6E, the amount of CD14 in lysosomes was significantly reduced in KO‐B1 iMG (Figure 7I). A similar trend was seen for KO‐F2 iMG (Figure S5G). Jointly, these cell biological analyses substantiated a role for SORLA in the sorting of CD14 between TGN, cell surface, and endo‐lysosomal compartments, a pathway crucial for pro‐inflammatory signal reception in microglia.

## 4 Discussion

We identified a unique role for SORLA in sorting of CD14 between cell surface and intracellular compartments in human microglia, trafficking paths central to the action of this pattern recognition receptor in the protection against CNS infection and injury (Janova et al. 2016). In our model, SORLA assists in sorting of CD14 through the TGN to the cell surface, priming microglia to pro‐inflammatory signal reception. Loss of SORLA activity results in aberrant accumulation of CD14 in the TGN, thereby reducing levels at the plasma membrane and blunting microglial responses to pro‐inflammatory stimuli at the cell surface and in endo‐lysosomal compartments.

SORLA is a risk factor for sporadic AD as well as a novel disease‐causing gene for the familial form of this disorder. Similar to other members of the VPS10P domain receptor gene family, SORLA acts as a sorting receptor directing cargo between TGN, cell surface, and endosomes (Malik and Willnow 2020). Earlier work by us and others has mainly focused on its role in protein sorting in neurons. In these studies, SORLA was shown to reduce amyloidogenic burden by two main mechanisms, by retrograde sorting of APP from endosomal compartments to the TGN to prevent precursor processing into Aβ (Dumanis et al. 2015; Herskowitz et al. 2012; Offe et al. 2006; Schmidt et al. 2007) and by anterograde sorting of newly produced Aβ from endosomes to lysosomes for degradation (Caglayan et al. 2014). While neuronal sorting functions of SORLA are features common to both human and rodent brains, expression and functional relevance of the receptor in microglia seems to be a distinguishing feature of the human brain (Gosselin et al. 2017; Hansen, Hanson, and Sheng 2018). This fact is in line with evidence from other areas in neuroscience where clear distinctions between human and murine microglia are seen, including our own work on glioma as a microglial‐relevant disease paradigm (Szulzewsky et al. 2016), necessitating novel humanized disease models of iPSC‐derived microglia (Hasselmann and Blurton‐Jones 2020; Huang et al. 2022).

Using unbiased transcriptomic analyses of SORLA‐deficient human microglia (iMG), we uncovered major changes in expression profiles related to endo‐lysosomal and vacuolar membrane compartments as well as the TGN (Figure 3C), supporting a role for SORLA in vesicular protein trafficking in microglia as well. This assumption is supported by the localization of the receptor to the TGN and endo‐lysosomal organelles in this cell type (Figure 3E). Comprehensive follow‐up phenotyping of WT and KO iMG failed to document a profound impact of receptor deficiency on basal cell migration or uptake of various compounds, including Aβ aggregates. Such sorting functions have been associated with the receptor in smooth muscle cells (McCarthy et al. 2010; Zhu et al. 2002) or neurons (Yajima et al. 2015), respectively. Rather, our findings identified a unique role for SORLA in microglial pro‐inflammatory responses as documented by global changes in inflammatory gene expression profile (Figure 3D) and by impaired release of pro‐inflammatory cytokines (Figure 5) in stimulated KO iMG as compared to WT. These defects were seen both with LPS and poly(I:C), arguing for a global role of the receptor in sensitizing microglia to various pro‐inflammatory signals. Interestingly, SORLA expression decreased following pro‐inflammatory stimulation, suggesting downregulation of receptor activity as a means to prevent overstimulation. A similar mechanism is operable for the homeostatic microglia gene P2RY12 (Suzuki et al. 2020), supporting a physiological function for SORLA in homeostatic microglia, as recently proposed by comprehensive mapping of human microglia cell populations (Mancuso et al. 2024). Obviously, our data do not exclude that SORLA may impact other microglial functions not tested here, such as migration under guided conditions, as observed for TREM2‐dependent migration towards senile plaques (McQuade et al. 2020), or that (amyloid) particles used for uptake studies may have impacted our results as compared to a previous report (Liu et al. 2020). Still, a role in pro‐inflammatory signal reception emerges as an important receptor activity in this cell type, as deduced from the loss‐of‐function phenotypes seen in receptor‐deficient microglia in this study.

Although the exact nature of intracellular signaling pathway(s) impacted in mutant iMG have not been addressed in our study, SORLA's role in pro‐inflammatory stimulation of microglia likely stems from its universal action as sorting receptor, directing nascent or recycling CD14 molecules through the TGN to the cell surface. Such sorting paths are crucial steps in pro‐inflammatory stimulation of TLR3 and TLR4. In detail, CD14 moves double‐stranded (ds) RNA from the cell surface to endo‐lysosomal compartment for signaling by TLR3 (Lee et al. 2006). For TLR4, signaling in response to danger‐ and pathogen‐associated patterns (DAMPs and PAMPs), like LPS, occurs at the cell surface or following CD14‐dependent internalization to endosomes (Ciesielska, Matyjek, and Kwiatkowska 2021; Zanoni et al. 2011). Internalized CD14 recycles back to the plasma membrane for replenishment of surface levels, a path assisted by cytosolic adaptors SNX1, SNX2, and SNX6 that stabilize tubular subdomains for endosomal cargo recycling (Ciesielska et al. 2022). Intriguingly, the recycling of SORLA to the cell surface in neurons is facilitated by interaction with another member of this family of sorting nexins, called SNX27 (Huang et al. 2016). SNX27 acts as a cargo recognition adaptor in the retrograde sorting complex retromer (Steinberg et al. 2013).

Several recent studies reported an increase in the size of lysosomes in neurons and microglia lacking SORLA (Hung et al. 2021; Mishra et al. 2022; Mishra, Jayadev, and Young 2024). Using similar morphometric analyses based on immunostainings (Figure 3G) or automated imaging flow cytometry (Figure 7E–G), we failed to detect a pronounced impact of SORLA deficiency on lysosome area, lysosomal signal intensity, or lysosomal vesicle size. Still, we observed a trend towards slightly increased lysosome vesicle size in immunostainings that reached statistical significance in KO‐F2, but not KO‐B1 iMG (Figure 3G). While our data do not exclude subtle structural anomalies of lysosomes to contribute to CD14 missorting, our findings are most consistent with loss of SORLA‐mediated trafficking of CD14 through the TGN as the underlying molecular cause of a blunted response to LPS and poly(I:C). Our hypothesis is supported by immunoprecipitation of SORLA and CD14 (Figure 6F), indicating direct or indirect interaction of this pattern recognition co‐receptor with the SORLA sorting machinery. Our hypothesis is further supported by the depletion of CD14 from cell surface and lysosomal compartments in the absence of the sorting receptor (Figure 6C,E, Figure 7I). In KO cells, CD14 levels in the TGN increase, suggesting impaired TGN to cell surface sorting in the absence of SORLA (Figure 6D,E). This mechanism is reminiscent of SORLA's action in other cell types where it facilitates cell surface recycling of tropomyosin receptor kinase B (TrkB) in neurons (Rohe et al. 2013) or the insulin receptor in adipocytes (Schmidt et al. 2016).

Although our studies were conceived to identify physiological roles for SORLA in microglia, they also provide some food‐for‐thought concerning its mode of action as an AD risk factor in this cell type. Neuroinflammation is a hallmark of AD pathology with microglia playing central roles in mediating inflammatory responses in the diseased brain (Heneka, Kummer, and Latz 2014; Heppner, Ransohoff, and Becher 2015). Because of its ability to sensitize microglia to pro‐inflammatory stimuli, SORLA levels may well play a decisive role in shaping the neuroinflammatory milieu in the AD brain. Potentially, impaired SORLA activity, as seen with some AD‐associated *SORL1* variants (Holstege et al. 2023; Holstege et al. 2017; Holstege et al. 2022), may exacerbate pathology by reducing protective microglial immune responses. Conversely, increased expression with protective *SORL1* variants (Caglayan et al. 2012; Young et al. 2015) may promote neuroinflammatory processes by microglia, mitigating AD progression.

## Author Contributions

P.L.O., K.J.M., N.S.T., and V.S. designed and conducted the experiments and analyzed data. S.F. and H.K. established differentiation protocols and provided essential expert advice. H.R., E.L.L., A.R.K., and F.L.H. provided human specimens and performed neuropathological assessment of human brain tissues. P.L.O., K.J.M., H.K., S.D., and T.E.W. conceptualized the study and evaluated the data. P.L.O. and T.E.W. wrote the manuscript.

## Ethics Statement

Procedures involving human tissue and data were performed in accordance with the ethical standards of the respective institutional research committees and with the 1964 Helsinki Declaration and its later amendments or comparable ethical standards. Procedures were approved by the Scientific Ethics Committee for the Region of Midtjylland Denmark (De Videnskabsetiske Komitéer for Region Midtjylland, project no. M‐2017‐82‐17) or by the local ethics committee of Charité‐Universitätsmedizin Berlin (approval no. EA1/144/13). Autopsy and research consent were obtained prior to death from the donors or their representatives. Identities were encoded before sharing for sample processing or data analysis.

## Conflicts of Interest

The authors declare no conflicts of interest.

## Supporting information


**Data S1** Supporting Information.

## Data Availability

The data that support the findings of this study are available from the corresponding author upon reasonable request.
