# Supplementary material for: Alzheimer's Disease Risk Gene SORL1 Promotes Receptiveness of Human Microglia to Pro‐Inflammatory Stimuli
Source: Glia. 2024 Dec 17;73(4):857–72. doi: 10.1002/glia.24659 (PMC11845846; doi:10.1002/glia.24659)
Supplement: Supplementary file 1 — Data S1 Supporting Information. [file GLIA-73-857-s001.docx]

**SUPPLEMENTARY METHODS**

**Immunohistochemistry of human brain samples**

Parietal cortex tissue (1 cm^3^ cube) used for Fig. 1A was surgically removed and immediately incubated in artificial cerebrospinal fluid. One-hour post dissection, the brain tissue was transferred to 4% PFA in PBS at 4C° and incubated for 4 hours. The fixed tissue was washed in PBS and dehydrated in 30% sucrose in PBS for 24-48 hours at 4°C. Then, the tissue was mounted in OCT, cut on a cryostat to 12 µm sections, and stored on glass slides at -80°C.

Cortical brain tissue used for Fig. 1B-C was snap frozen in liquid nitrogen and cut to 5 µm fresh-frozen tissue sections. Sections were mounted on coverslips coated with 3-aminopropyltriethoxysilane (APES) and stored at -80°C for 1-2 weeks before immunostaining. Before immunostaining procedure, the tissue was post-fixed in 4% PFA in PBS for 10 min and washed in PBS.

For all immunostainings, cryosections of human brain tissues were washed in PBS, incubated with blocking buffer (5% normal donkey serum, 0.1% Triton X-100 in PBS) at room temperature for 30 min, followed by incubation with primary antibodies in blocking buffer at 4°C overnight. The next day, sections were washed in PBS, incubated with secondary antibodies in blocking buffer (1:500) for 2 hours at room temperature, counterstained with DAPI (1:3000, 10236276001, Roche), and mounted in fluorescent mounting media (DAKO). Used primary antibodies were in-house goat anti-SORLA and rabbit anti-SORLA (kindly provided by C. Munch Petersen, Aarhus University), as well as commercial antibodies directed against IBA1 (ab5076, Abcam), P2RY12 (HPA014518, Sigma), and TMEM119 (HPA051870, Sigma). Alexa Fluor 488 (Jackson ImmunoResearch, 711-545-152), 555 (Invitrogen, A32816), and 647 (Invitrogen, A31571) were used as secondary antibodies.

**Analysis of pluripotency**

Pluripotency and the ability to generate­ all three germ layers were assessed by TaqMan Scorecard Assay (Applied Biosystems) using iPSCs and spontaneously differentiated embryoid bodies (EB) as described (Marczenke et al., 2021). In brief, iPSCs were grown to 70-80% confluency and collected in lysis buffer for RNA purification using RNeasy Mini kit (Qiagen, USA). For EB formation, iPSCs were dissociated with accutase, seeded in Nucleon Sphera 96-well U-shaped-bottom plates (Thermo Fisher Scientific) at a density of 8000 cells/well in 150 µl E8 medium, supplemented with 10 μg/ml Y27632, and centrifuged for 3 minutes at 200x g. The next day, 150 µl EB medium (DMEM/F12 (31330-038, Gibco), supplemented with 10% KnockOut Serum Replacement (10828010, Gibco), 1x GlutaMAX (35050061, Thermo Fisher Scientific), 1x NEAA (11140050, Thermo Fisher Scientific), and 1% penicillin-streptomycin (Gibco)), was added to each well. Thereafter, the medium was changed every second day. At day 7, a pool of 20 EBs per genotype were collected in lysis buffer for RNA purification using RNeasy Mini kit (Qiagen, USA). The scorecard assay was performed by qRT-PCR on total RNA isolated from iPSCs (day 0) and EBs (day 7) according to the manufacturer’s protocols. Gene expression data were analyzed using the web-based hPSC Scorecard Analysis Software (Thermo Fisher Scientific).

**Expression analyses**

Transcript levels in cultured iPSC and iMG were analyzed in total RNA isolated using the RNeasy Plus Micro kit (Qiagen, USA) and reverse transcribed to cDNA with high-capacity RNA to cDNA kit (Applied Biosystems, USA). The cDNA samples were subjected to quantitative (q) RT-PCR using TaqMan Gene Expression Assays *SOX2* (Hs01053049_s1), *OCT4* (Hs00999632_g1), *NANOG* (Hs02387400_g1), *SORL1* (Hs00983770), *IBA1* (Hs00610419), *P2RY12* (Hs01881698), *CX3CR1* (Hs01922583_s1), *TREM2* (Hs00219132_m1), *HPRT1* (Hs02800695_m1), *GAPDH* (Hs02758991_g1), *TBP* (Hs00427620_m1). Relative gene expression was quantified with the cycle threshold (CT) comparative method (2^-ddCT^). Data were normalized to reference genes *GAPDH*, *HPRT1*, and *TBP* and fold change relative to levels in iPSC or the WT control were determined as stated in the respective figure legends.

Total protein expression levels were evaluated by standard Western blot analysis procedures using primary and horseradish peroxidase-conjugated secondary antibodies (Sigma-Aldrich) and the SuperSignal West Femto Maximum Sensitivity Substrate (Thermo Fisher Scientific). Primary antibodies were mouse anti-SORLA (BD611861, BD Biosciences), mouse anti-GAPDH (gtx627408, GeneTex), and rabbit anti-CD14 (CS56082, Cell Signaling Technology).

**Immunocytochemistry**

Induced microglia (iMG) were seeded on non-coated 12 mm glass coverslips in a 24-well plate and allowed to attach overnight. The next day, cells were washed in PBS once and fixed with 4% PFA in PBS for 10 min at room temperature. Fixed cells were washed twice in PBS and stored in 0.02% NaAzide in PBS at 4°C until further use. Immunostainings were performed as described for brain tissue above. Primary antibodies were in-house goat and rabbit anti-SORLA antisera, as well as commercially available antibodies directed against IBA1 (ab5076, Abcam), P2RY12 (HPA014518, Sigma), EEA1 (2411S, Cell Signaling), EEA1 (610457, BD Biosciences), GM130 (ab52649, Abcam), TGN46 (AHP500GT, Biorad), LAMP1 (9091, Cell Signaling), LAMP1 (ab24170, Abcam), CD14 (ab181470, Abcam). Co-localization of immunostained SORLA and CD14 and subcellular compartment markers were analyzed by Pearson's and Mander´s correlation coefficient respectively using FIJI (ImageJ) and the BIOP´s (Bioimaging and Optics Platform) version of JaCoP (Just Another Colocalization Plugin) (Bolte & Cordelières, 2006). Measurements of EEA1 and LAMP1 area and particle size were performed using FIJI (ImageJ) analyze particles function.

Measurements of EEA1 and LAMP1 area and particle size were performed using FIJI (ImageJ).

**Scratch wound assay**

Induced MG were seeded in 96-well ImageLock plates (Sartorius) at a density of 100.000 cells per well and incubated overnight. Scratches were made using Woundmaker (Essen Bioscience), followed by a full medium change with fresh microglia differentiation medium to remove cell debris. Cell plates were transferred to an Incucyte SX5 live imaging system (Sartorius) and phase contrast images acquired every hour for 24 hours at 10x magnification. Images were analyzed using the scratch wound analysis tool (Incucyte) and presented as percentage relative wound density. In brief, an AI-based cell detection program defined the wounded area at t = 0 hour and compared the cell confluency within the wounded area (in %) with the non-wounded area over time. Confluency within the wounded area was normalized to 0% at t = 0 hour.

**Phagocytosis assays**

Zymosan Green pHrodo (P35365, Invitrogen) and *E. coli* Red pHrodo (P35361, Invitrogen) were dissolved in PBS at 1 mg/ml. To homogeneously disperse the particles, the solutions were triturated with a p1000 pipette and vortexed for 30 sec, followed by sonication for 20 min using a water bath sonicator. Aliquots were stored at -80°C until further use. Before use, aliquots were sonicated for 10 min and vortexed briefly. HiLyte™ Fluor 488-labeled amyloid-β 1-42 (AS-60479-01, AnaSpec) was used to generate oligomeric and fibrillar aggregates according to a previous protocol (Stine et al., 2011). In brief, a solution of soluble Aβ was generated by reconstituting the lyophilized peptide in 1% NH_4_OH at 10 mg/ml. After vigorous pipetting, PBS was added to reach a final stock concentration of 0.5 mg/ml. The solution was resuspended by pipetting and quickly aliquoted for storage at -80°C. All steps were performed on ice with ice-cold reagents to avoid aggregation. Oligomeric and fibrillary Aβ batches were generated by incubating aliquots for 18 hours at 4°C or 37°C, respectively.

For the phagocytosis assays, iMGs were seeded in 96-well plates (Corning) at a density of 50.000 cells per well and incubated overnight. The next day, the medium was replaced with fresh microglia differentiation medium containing fluorescent particles at a concentration of 2 µg/ml. Uptake of fluorescent particles was imaged using an Incucyte SX5 fluorescence live imaging system. Images were acquired every hour for 24 hours at 20x magnification. Cellular uptake of fluorescent particles was analyzed using the basic analysis tool (Incucyte) and presented as ratio of fluorescence area relative to the total cell area.

**Pro-inflammatory response**

Induced MG were seeded in 96-well plates at a density of 50.000 cells per well and incubated overnight. The next day, the medium was replaced with 100 μl microglia differentiation medium containing PBS (control), 100 ng/ml lipopolysaccharide (LPS) from *E.coli* O111:B4 (L4391, Sigma), or 10 μg/ml polyinosinic-polycytidylic acid (poly(I:C)) (IAX-200-021, Innaxon). After 24 hours of incubation, media samples were collected for multiplex immunoassay biomarker analysis (Olink proteomics) or targeted ELISA.

A multiplex immunoassay panel (Target 96 Inflammation, Olink Proteomics, Uppsala, Sweden), using the proximity extension technique, was used to analyze 92 inflammatory related biomarkers as described elsewhere (Schram et al., 2022). The analysis was performed using BioMark™ HD, Fluidigm® at BioXpedia A/S, Aarhus, Denmark. In brief, 1 μl iMG-conditioned medium was incubated with 92 oligonucleotide-labeled antibody pairs for proximity extension and quantification by standard qRT-PCR reaction. A blank media sample was used to define background signals. The proximity extension assay readout was normalized to protein expression units (NPX) using IPC normalization. For the differential expression analyses, samples were first checked for normal distribution by Shapiro-Wilk followed by Student t-test or Wilcoxon rank sum test. Multiple testing was corrected for using the Benjamini-Hochberg method.

The following ELISA kits were used to determine target cytokine levels in media samples from iMG: human TNFα, IL1β, IL6, IL10, IL18, RANTES (K15231N-1, K15067L-1, U-Plex, Meso Scale Discovery), human IFN-ß (151ADRS-1, S-Plex, Meso Scale Discovery), and human CD14 (Quantikine ELISA Kit (DC140, R&D). Assays were performed according to the manufacturers’ protocols.

**Immunoprecipitation**

HEK293 cells were cultured in DMEM (41966-029, Gibco) containing 10% fetal bovine serum and passaged several times before being seeded into 6-well plates. At 50% confluency, the cells were transfected with expression constructs for SORLA (pcDNA3.1-hSORL1) (Schmidt et al., 2007) and/or CD14 (pCMV6-hCD14-Myc-DKK, RC229962, Origene) using Lipofectamine 2000 (11668030, Invitrogen). Forty-eight hours after transfection, the cells were lysed for 1 hour on ice in IP lysis buffer (150 mM NaCl, 25 mM Tris-HCl pH 7.4, 1 mM EDTA, 1% NP40, 5% glycerol), supplemented with complete Protease Inhibitor Cocktail (Roche). The Pierce BCA Protein Assay Kit (Thermo Fisher Scientific) was used to determine protein concentrations. Thirty μg of protein lysate were used as input control and 500 μg protein lysate for co-immunoprecipitation (co-IP). Co-IP was performed using anti-DYKDDDDK Magnetic Agarose beads (A36797, Thermo Fisher Scientific) for IP of CD14 or in-house goat anti-SORLA coupled to Protein G-agarose beads (11243233001, Sigma) for IP of SORLA. Protein-bound beads were washed in buffer (150 mM NaCl, 25 mM Tris-HCl pH 7.4, 0.05% Tween-20) followed by elution of bound proteins in 2x Laemmli sample buffer (112 mM Tris, 16.2% Glycerol, 4.2% SDS 5% ß-Mercaptoethanol) for 5 min at 96°C. Input control and co-IP samples were subjected to standard Western blotting.

**SUPPLEMENTARY REFERENCES**

Bolte, S., & Cordelières, F. P. (2006). A guided tour into subcellular colocalization analysis in light microscopy. *Journal of Microscopy*, *224*(Pt 3), 213–232. https://doi.org/10.1111/j.1365-2818.2006.01706.x

Marczenke, M., Sunaga-Franze, D. Y., Popp, O., Althaus, I. W., Sauer, S., Mertins, P., Christ, A., Allen, B. L., & Willnow, T. E. (2021). GAS1 is required for NOTCH-dependent facilitation of SHH signaling in the ventral forebrain neuroepithelium. *Development (Cambridge, England)*, *148*(21). https://doi.org/10.1242/dev.200080

Schmidt, V., Sporbert, A., Rohe, M., Reimer, T., Rehm, A., Andersen, O. M., & Willnow, T. E. (2007). SorLA/LR11 regulates processing of amyloid precursor protein via interaction with adaptors GGA and PACS-1. *The Journal of Biological Chemistry*, *282*(45), 32956–32964. https://doi.org/10.1074/jbc.M705073200

Schram, A.-S. L., Sellmer, A., Nyboe, C., Sillesen, M., & Hjortdal, V. E. (2022). Increased inflammatory markers in adult patients born with an atrial septal defect. *Frontiers in Cardiovascular Medicine*, *9*, 925314. https://doi.org/10.3389/fcvm.2022.925314

Stine, W. B., Jungbauer, L., Yu, C., & LaDu, M. J. (2011). Preparing synthetic Aβ in different aggregation states. *Methods in Molecular Biology (Clifton, N.J.)*, *670*, 13–32. https://doi.org/10.1007/978-1-60761-744-0_2

**SUPPLEMENTARY FIGURES**

**
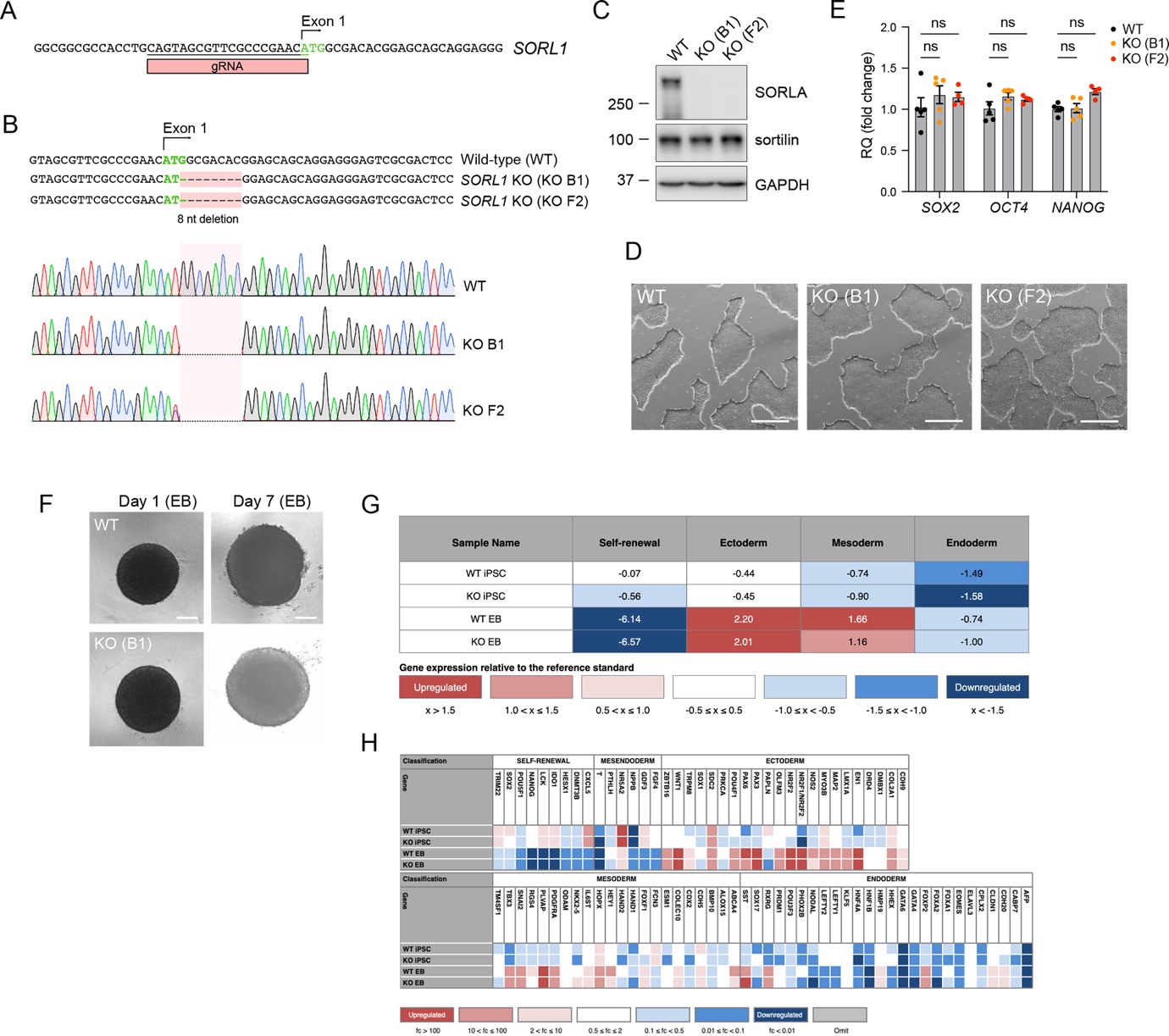
**

**_­­_**

**Supplementary figure 1. Validation of wildtype and SORLA-deficient human induced pluripotent stem cell lines**

**(A, B)** CRISPR/Cas9 strategy used to disrupt *SORL1* in the human induced pluripotent stem cell (iPSC) line HMGUi001-A. Placement of the guide RNA (gRNA) at the ATG start codon of the wildtype (WT) *SORL1* locus is shown in A. Comparative genome sequence analyses of *SORL1* loci in WT and two independent SORLA-deficient cell clones (KO-B1 and KO-F2) are depicted in B, documenting the same 8 nucleotide (nt) deletion at the start codon in both lines. **(C)** Western blot analysis of SORLA and sortilin expression in lysates of WT and KO (B1, F2) iPSC lines. Detection of GAPDH was used as loading control. The migration of protein marker bands of indicated molecular weights (in kDa) are given. **(D)** Phase contrast images of WT and KO (B1, F2) iPSC lines. Scale bar: 500 µm. **(E)** Levels of transcripts for pluripotency markers *SOX2*, *OCT4*, and *NANOG* in WT and KO (B1, F2) iPSC lines as determined by qRT-PCR. Relative quantification (RQ) fold change represents 2^-ddCt^ normalized to WT levels (set to 1). *GAPDH*, *HPRT1*, and *TBP* were used as reference genes (WT, n=5; KO-B1, n=5; KO-F2, n= 4 biological replicates). Statistical significance of data was determined using Two-Way ANOVA with repeated measures and corrected for multiple testing by Dunnett. **(F**) Phase contrast images of WT and KO-B1 embryoid bodies (EB) at day 1 and day 7 of culture. Scale bars: 200 µm. (**G**) Quantitative RT-PCR based score card assay documents similar scores for self-renewal as well as ectoderm, mesoderm and endoderm differentiations for WT and KO-B1 iPSC lines and EBs when compared to the expression profiles of the reference standard. **(H)** Heatmaps of analyzed genes related to self-renewal, mesendoderm, ectoderm, mesoderm, and endoderm fates in WT and KO-B1 iPSC lines, and EB derived thereof. Values and colors correlate to the fold changes (FC) of each gene relative to the undifferentiated reference set.

**
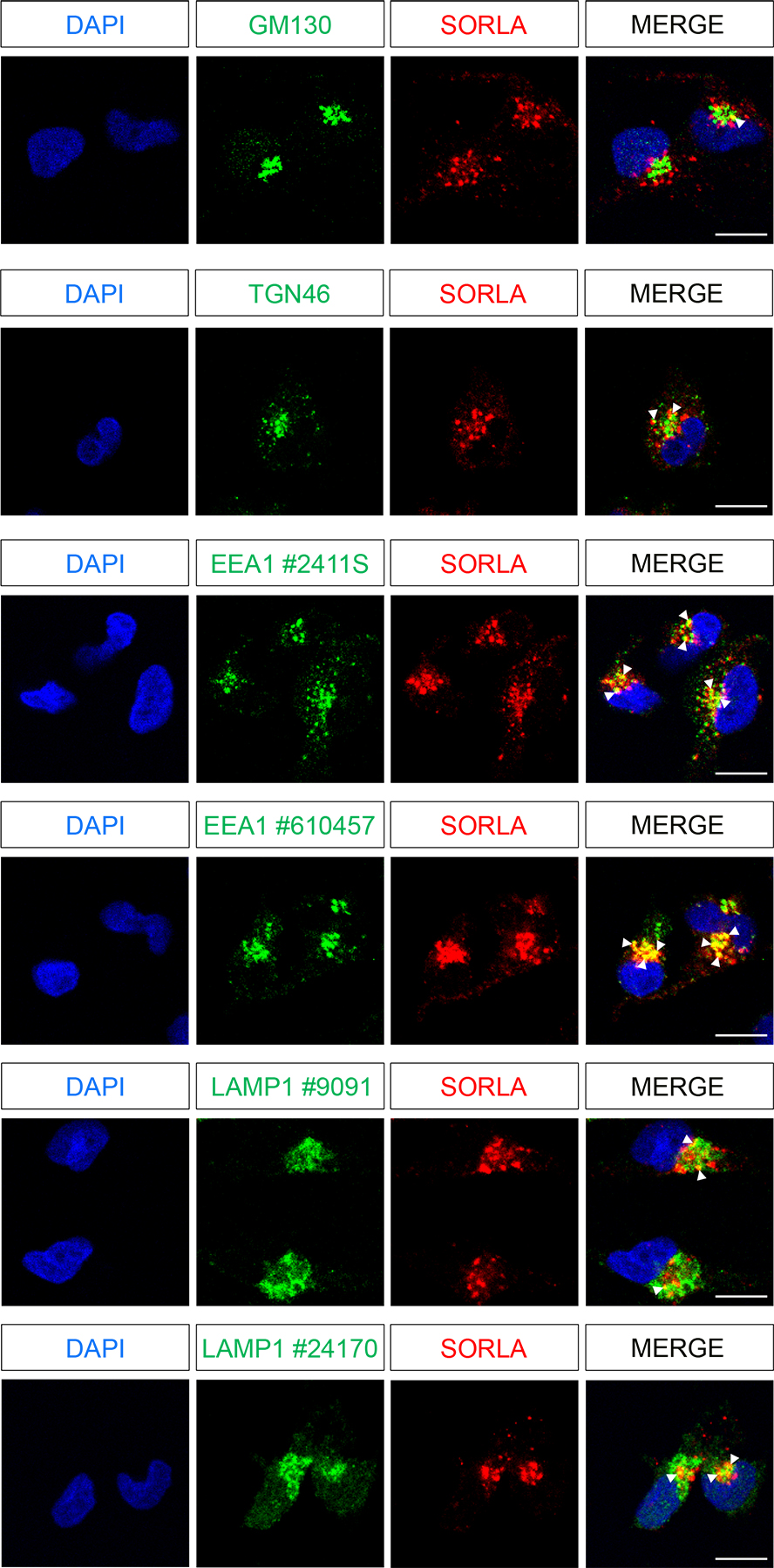
**

**Supplementary figure 2. Subcellular localization of SORLA in wildtype human iMG**

Immunofluorescence detections of SORLA (red) and the indicated markers of subcellular compartments (green) in iMG are shown. DAPI: nuclei, GM130: *cis-*Golgi, TGN46: trans-Golgi Network (TGN), EEA1: early endosomes, LAMP1: lysosomes. White arrowheads exemplify colocalization of SORLA with EEA1 or LAMP1 in the respective merged images. To substantiate accuracy of the organelle patterns, two different antibodies each directed against EEA1 or LAMP1 were used (suppliers’ catalogue numbers as stated in the method section). Images of 60-100 cells per condition were used for Pearson´s correlation coefficient analysis of subcellular co-localization of SORLA shown in Fig. 3E. Scale bars: 10 µm.

**
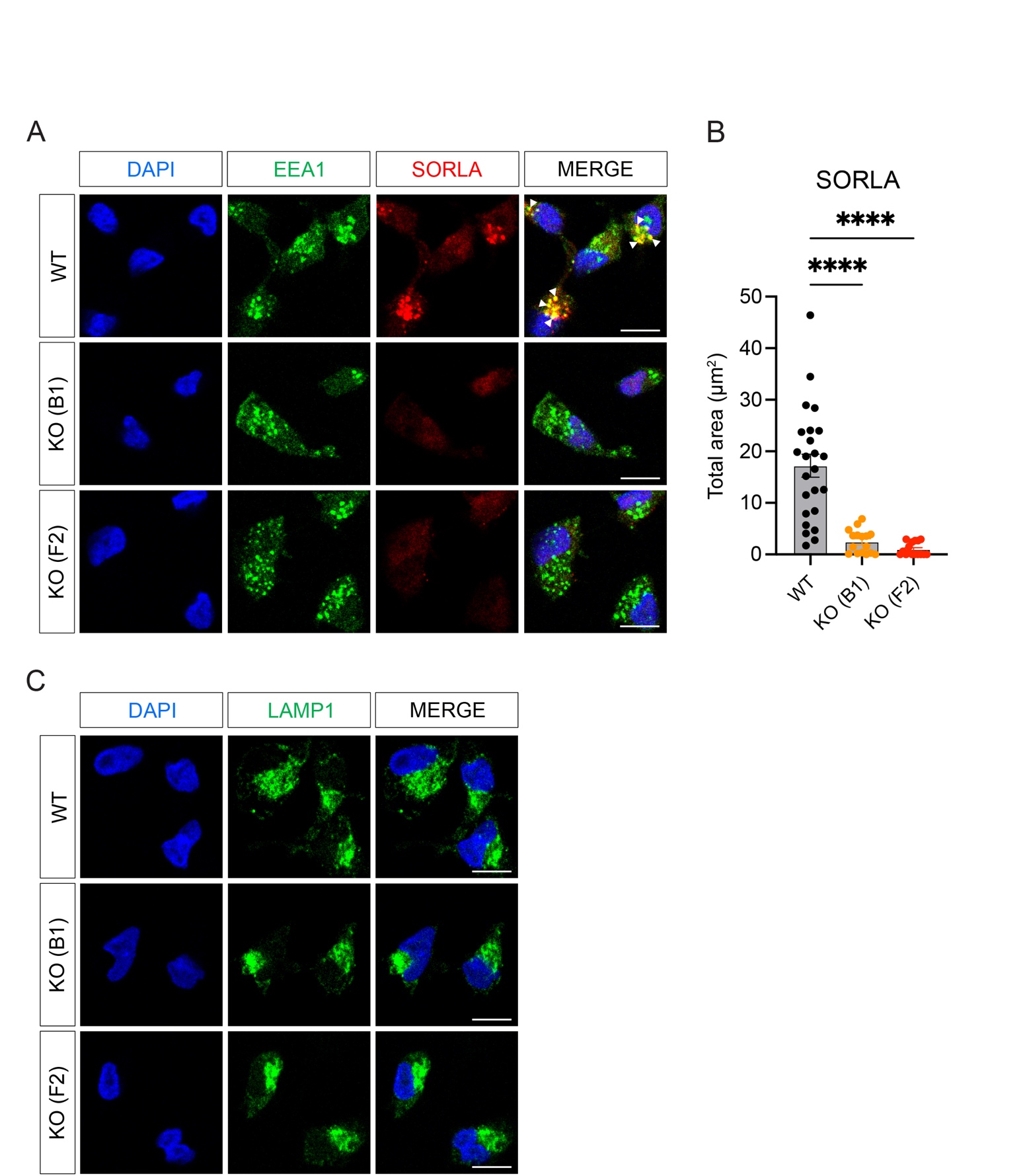
**

**Supplementary figure 3. Immunodetection of endo-lysosomal compartments in WT and SORLA-deficient human iMGs**

Immunofluorescence detection of SORLA and EEA1 (**A**) or LAMP1 (**C**) in wildtype and SORLA-deficient iMG lines (clones KO-B1 and KO-F2). Scale bars: 10 µm. (**B**) Quantitative analysis of SORLA signal intensities from panel A, documenting complete absence of receptor immunoreactivity in KO-B1 and KO-F2 iMG (WT, n=24; KO-B1, n=16; KO-F2, n=16 cells from two independent experiments). Statistical significance of data was determined using One-Way ANOVA corrected for multiple testing by Dunnett. Images of 15-24 cells from two independent differentiation experiments, as exemplified in A and C, were used for morphometric analyses of endosomal (EEA1^+^) and lysosomal (LAMP1^+^) compartments given in Fig. 3F and G.

**
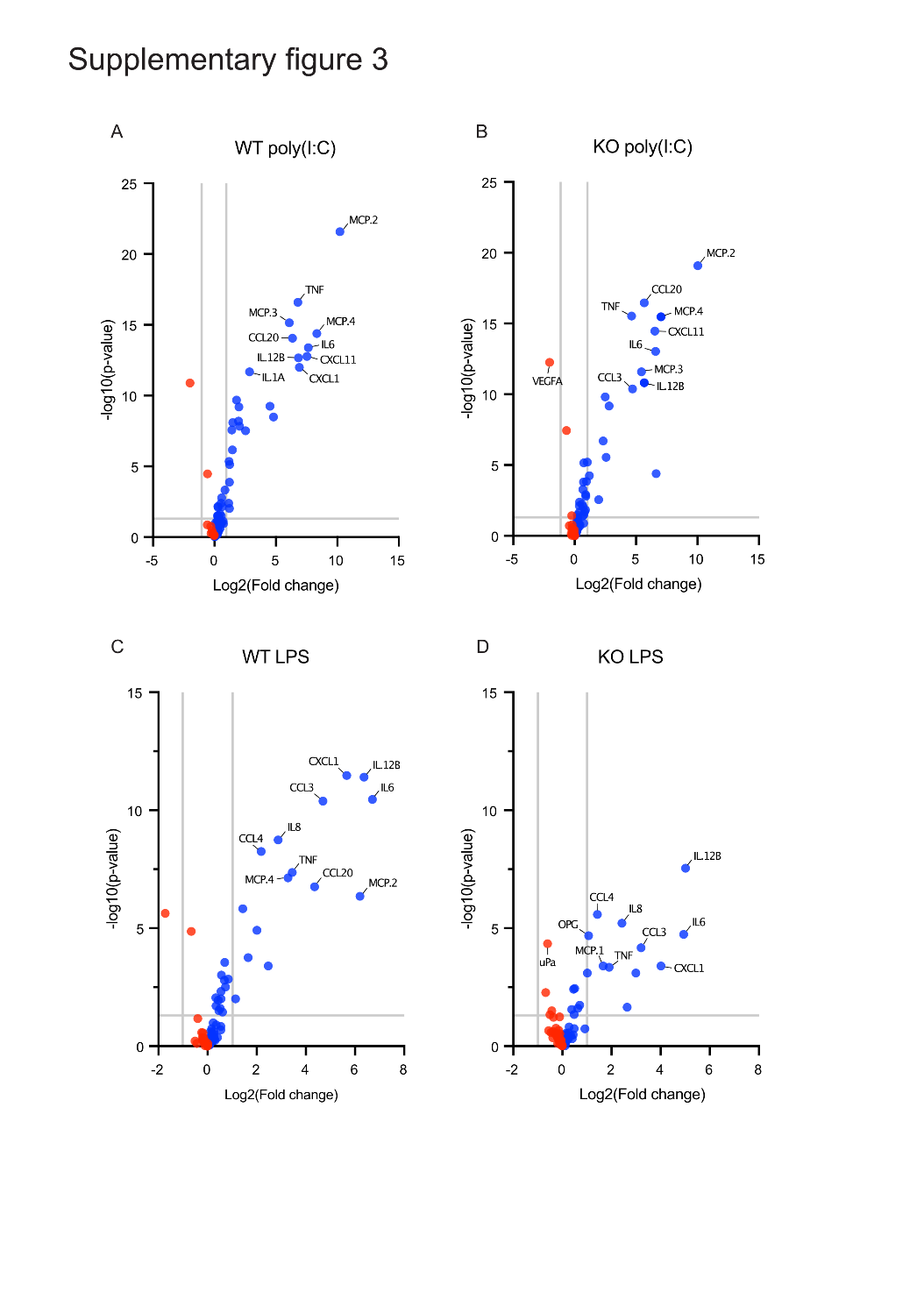
**

**Supplementary figure 4: Effects of pro-inflammatory stimulation in WT and SORLA-deficient iMG**

Volcano blots of differentially expressed inflammatory markers based on multiplex immunoassays (illustrated in Fig. 5B-C) with log2(fold change) and -log10(p-value). Red and blue dots indicate molecules that are down- or up-regulated, respectively, in supernatants of WT (A, C) or KO-B1 (B, D) iMG treated with 10 µg/ml poly(I:C) (A, B) or 100 ng/ml LPS (C, D) for 24 hours compared with unstimulated (PBS) genotype controls. Grey horizontal and vertical lines represent non-adjusted p-values equal to 0.05 and log2(fold change) of -1 and 1, corresponding to a halving or doubling in protein levels, respectively. The top 10 proteins with p-value < 0.05 are marked in the blots.

**
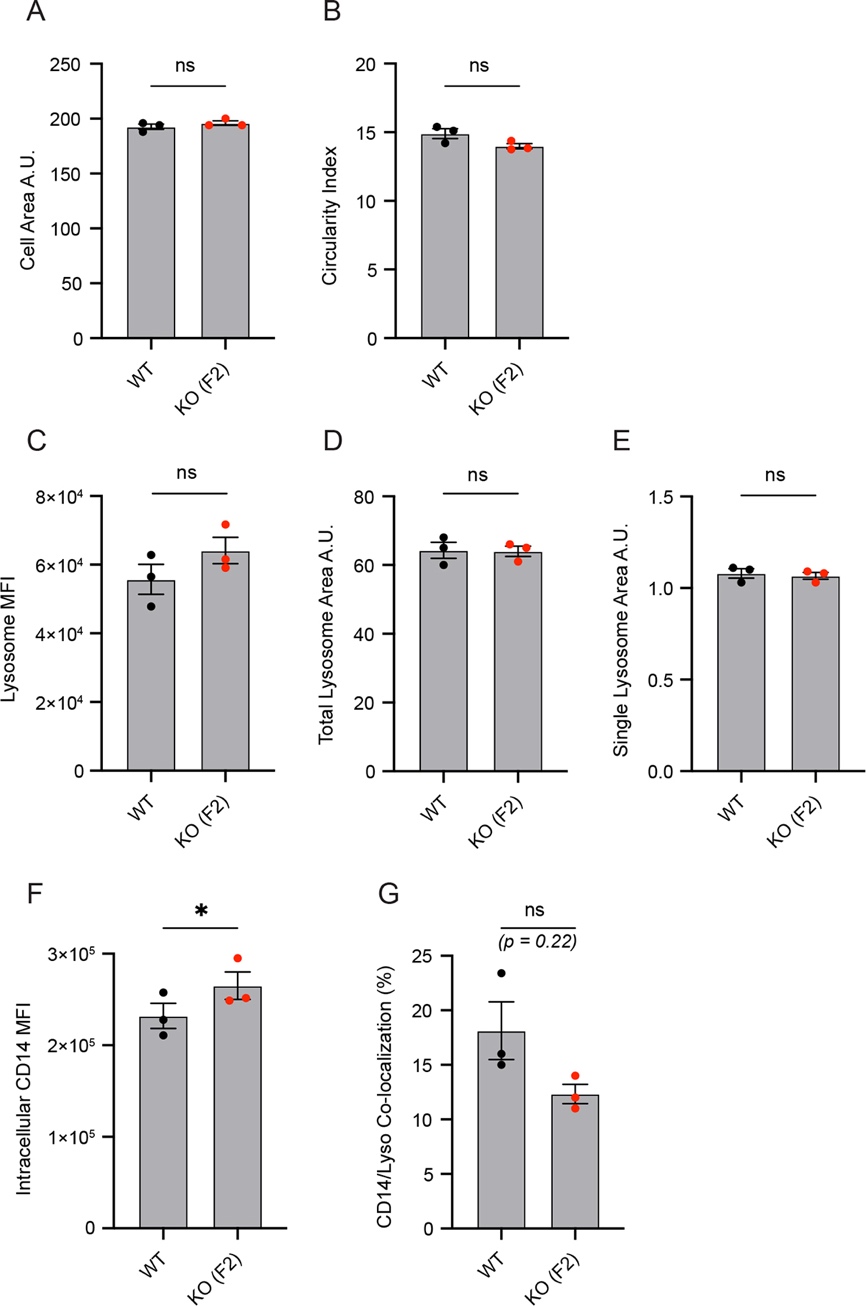
**

**Supplementary figure 5: SORLA controls intracellular distribution of CD14 in iMG**

Intracellular distribution of CD14 in WT and KO-F2 iMG was analyzed using imaging flow cytometry as detailed in the legend to Fig. 7 (n=3 biological replicates, each replicate representing 10,000 cells tested). **(A, B)** Quantification of cell size (A; in arbitrary units, A.U.) and cell shape (B; circularity index) in WT and KO-F2 iMG based on the full cell mask. **(C-E)** Quantification of intracellular lysotracker signal was used to determine total mean fluorescence intensity of lysosomes (MFI; C), total lysosome area (D), as well as lysosome vesicle size (E). **(F)** Quantification of intracellular CD14 signal as determined by MFI. **(G)** Quantification of co-localization between intracellular CD14 and lysotracker signals, given as % of CD14 signal located in lysotracker^+^ areas. Statistical significance of data was determined using paired Student’s t-test.
